# The dynamic state of a prefrontal–hypothalamic–midbrain circuit commands behavioral transitions

**DOI:** 10.1038/s41593-024-01598-3

**Published:** 2024-03-18

**Authors:** Changwan Chen, Mahsa Altafi, Mihaela-Anca Corbu, Aleksandra Trenk, Hanna van den Munkhof, Kristin Weineck, Franziska Bender, Marta Carus-Cadavieco, Alisa Bakhareva, Tatiana Korotkova, Alexey Ponomarenko

**Affiliations:** 1 Max Planck Institute for Metabolism Research, Cologne, Germany; 2 Institute for Systems Physiology, Faculty of Medicine, University of Cologne/University Clinic Cologne, Cologne, Germany; 3 Institute of Physiology and Pathophysiology, Friedrich-Alexander-Universität Erlangen-Nürnberg, Erlangen, Germany; 4 Department of Neurophysiology and Chronobiology, Institute of Zoology and Biomedical Research, Jagiellonian University, Krakow, Poland; 5 Behavioural Neurodynamics Group, Leibniz Institute for Molecular Pharmacology (FMP)/NeuroCure Cluster of Excellence, Berlin, Germany; 6 Excellence Cluster on Cellular Stress Responses in Aging Associated Diseases and Center for Molecular Medicine Cologne, University of Cologne, Cologne, Germany

**Keywords:** Neural circuits, Social behaviour, Hypothalamus, Cognitive control, Prefrontal cortex

## Abstract

Innate behaviors meet multiple needs adaptively and in a serial order, suggesting the existence of a hitherto elusive brain dynamics that brings together representations of upcoming behaviors during their selection. Here we show that during behavioral transitions, possible upcoming behaviors are encoded by specific signatures of neuronal populations in the lateral hypothalamus (LH) that are active near beta oscillation peaks. Optogenetic recruitment of intrahypothalamic inhibition at this phase eliminates behavioral transitions. We show that transitions are elicited by beta-rhythmic inputs from the prefrontal cortex that spontaneously synchronize with LH ‘transition cells’ encoding multiple behaviors. Downstream of the LH, dopamine neurons increase firing during beta oscillations and also encode behavioral transitions. Thus, a hypothalamic transition state signals alternative future behaviors, encodes the one most likely to be selected and enables rapid coordination with cognitive and reward-processing circuitries, commanding adaptive social contact and eating behaviors.

## Abstract (web summary)

Chen et al. show that transitions to innate behaviors, such as feeding and social interaction, rely on their encoding during beta oscillations by neuron populations in the lateral hypothalamus, coordinated with the medial prefrontal cortex and ventral tegmental area.

## Main

In humans and other mammals, hypothalamic neurons change their activity during innate behaviors^1,2^, which can be elicited or inhibited by the activation of genetically identified hypothalamic cell populations^1,3–6^. The lateral hypothalamus (LH) features particularly strong efferent and afferent connections with multiple forebrain regions compared to other hypothalamic outputs^7^. Afferents from the forebrain, which transmit information about sensory cues, previous experience and brain state, are thought to interact with metabolic, visceral and hormonal signals in the hypothalamus, resulting in the selection of a hypothalamic cellular activity output that is congruent with the adaptively relevant behavior^8^. These computations closely resemble the brain control mechanism for switching to a new instinctive behavior proposed in seminal ethological studies^9^. However, the neurodynamic underpinnings of hypothalamic information processing during behavioral transitions remain unknown.

At timescales of milliseconds to tens of milliseconds, hypothalamic cells fire with varying probabilities depending on the phase of network oscillations^10,11^. In sensory, associative cortical areas and the hippocampus, oscillations enable the separate signaling of different sensory or behavioral events at distinct times of oscillation cycles, a phenomenon known as phase coding^12–14^. This form of temporal coding is thought to facilitate the communication between cells firing together and strengthen their influence in neural circuits involved in memory acquisition and retrieval, as well as sensory processing^15,16^.

In this study, we show that the activity of neuronal populations in the LH at specific phases of beta (15–30 Hz) oscillations encodes transitions to innate behaviors, namely feeding (F), social contact (S) or exploration of a new object (E). We found that the neuronal discharge in the LH and the lateral preoptic area (LPO) is coordinated with a phase offset from transition populations. An optogenetic manipulation of this offset, changing the timing of LPO inhibition of the LH, eliminated behavioral transitions. We further show that the regulation of LH neuronal activity by inputs from the medial prefrontal cortex (mPFC) promotes behavioral transitions and demonstrate that beta oscillations contribute to signaling between the LH and the ventral tegmental area (VTA), where putative dopaminergic cells display behavior-specific activity during transitions between innate behaviors.

## Results

### Firing rate of LH neurons and beta oscillations in a free-choice model

**Fig. 1:**
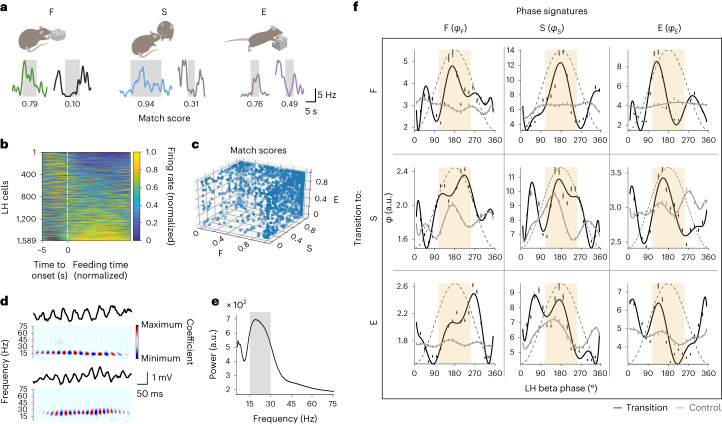
Representations of innate behaviors during beta oscillations in the LH. **a**, Examples of behavior-dependent firing rate changes in LH cells. Increasing (high match score cells) or decreasing (low match score cells) firing rate during feeding (F), social contact (S) and new object exploration (E). The gray shading represents the respective behaviors. **b**, Feeding-dependent changes in the firing rate of LH cells. Feeding onset is represented by the white dashed line. The duration of the episodes was uniformly scaled; *n* = 1,589 cells from seven mice. **c**, Distribution of the firing rate match scores of LH cells for F, S and E; *n* = 1,363 cells from seven mice. **d**, Representative traces of LFP beta oscillations in the LH (top, 1–100 Hz band pass) and their wavelet spectrograms (bottom, scalograms) in freely moving mice (see Extended Data Fig. 1d for further traces, recorded during transitions to individual behaviors). **e**, An average power spectrum of beta oscillations detected in a representative LFP recording in the LH; mean ± s.e.m. of 2,059 oscillation envelopes. The shaded area represents the beta frequency band. a.u., arbitrary unit. **f**, Phase signatures during transitions to behaviors (bootstrap) and during 1,000 shuffled sets of control epochs excluding these transitions; mean ± 95% confidence interval (CI); polynomial fits. The columns represent the behavioral phase signatures related to F (*φ*_F_), S (*φ*_S_) and E (*φ*_E_). The rows represent the transition to: F, *n* = 110 cells; S, *n* = 420 cells; and E, *n* = 292 cells from five mice. Peak neighborhood phases are defined as four bins (72°) flanking the oscillation peak (ivory shade); the dashed sine curve represents the reference cycle. See also Supplementary Information for the statistical information related to **a**–**c**. Source data

**Extended Data Fig. 1:**
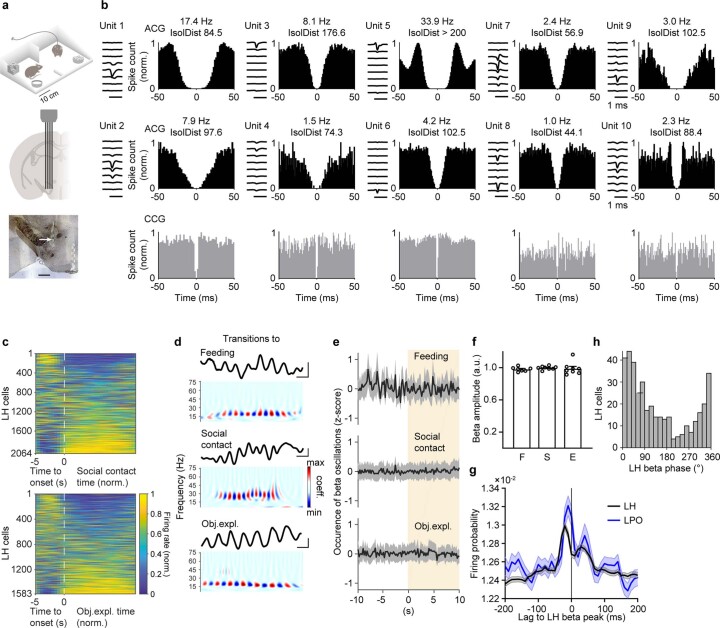
Neuronal discharge and beta oscillations in the LH. **a**, Top: enclosure with a free access to food, water, novel object and a freely moving conspecific. Middle: chronic recordings in the LH using a movable silicon probe. Bottom: a representative histological section showing a probe track in the LH. White arrow, localization of an electrolytic lesion. Dashed vertical line, the 3rd ventricle. Scale bar, 200 μm. **b**, Representative single units sorted in silicon probe recordings from the LH. Average spike waveforms from eight channels of the probe’s shank (scale bars, 1 ms), ACGs (black, firing rate and isolation distance are indicated for each unit) and CCGs (grey) of the shown units recorded by the same shank (columns). Bin width 1 ms. **c**, Behavior-dependent changes of LH cells’ firing rate. Behavior onset: dashed line, episodes’ duration is uniformly scaled. Top: social contact, n = 2064 cells from eight mice; bottom: obj.expl. (novel object exploration), n = 1583 cells from eight mice. **d**, Representative traces of LFP beta oscillations (scale bars: 50 ms; 0.5 mV) in the LH (1 - 100 Hz band-pass) during transitions to feeding, social contact and obj.expl., and their wavelet spectrograms. **e**, CCGs between beta oscillation times (maximal amplitude of envelopes) and onsets of feeding, N = 7 mice, social contact and obj.expl., N = 8 mice. **f**, The amplitude of beta oscillations during each behavior normalized to the amplitude during control epochs not including the respective behavior, feeding vs. control, N = 7 mice, p = 0.09, social vs. control, N = 8 mice, p = 0.8, obj.expl. vs. control, N = 8 mice, p = 0.7, t-test. **g**, The probability of LH (n = 2417 cells from eight mice) and LPO (n = 415 cells from three mice) neuronal discharge in relation to the maximum amplitude of LH beta oscillation envelopes. **h**, Histogram of preferred LH beta oscillation phases for significantly modulated LH cells, n = 384 cells from eight mice. Data are presented as mean ± SEM. See also Supplementary Information for the statistical information for **c**, **e**, **f**. Source data

Using movable silicon probes, we recorded the firing of 2,417 LH neurons while mice were engaging in F, S or E in a free-choice model (Fig. 1a and Extended Data Fig. 1a,b). As reported previously^1,17^, individual LH neurons increased or, less often, decreased their firing rate during episodes of each of the three behaviors compared to other recording epochs (Fig. 1a,b and Extended Data Fig. 1c). The changes in firing rate were estimated as a match score, computed for each cell and behavior, with higher match scores corresponding to an increase of the firing rate relative to time-shuffled epochs (Fig. 1a,c).

Across behaviors, the LH local field potential (LFP) displayed fast oscillations, with the leading frequency in the beta band (15–30 Hz) for approximately 26% of the recording time (Fig. 1d,e and Extended Data Fig. 1d). Beta oscillations are part of the synchronization repertoire of cortical and basal ganglia networks where they are thought to support sensory, motor and short-term memory processing in different brain circuits^15,18–20^. While beta oscillation episodes lasting 202 ± 3 ms (*n* = 8 mice) occurred at a similar rate around behavioral transitions and had similar amplitudes across behaviors (Extended Data Fig. 1e,f), they were associated with marked changes in the firing of LH neurons. The firing probability of LH neurons increased around the times when the amplitude of beta oscillations was the highest (Extended Data Fig. 1g). A substantial fraction of LH neurons was entrained by beta oscillations (21%, Rayleigh test); most were phase-locked to oscillation troughs (Extended Data Fig. 1h).

**Extended Data Fig. 2:**
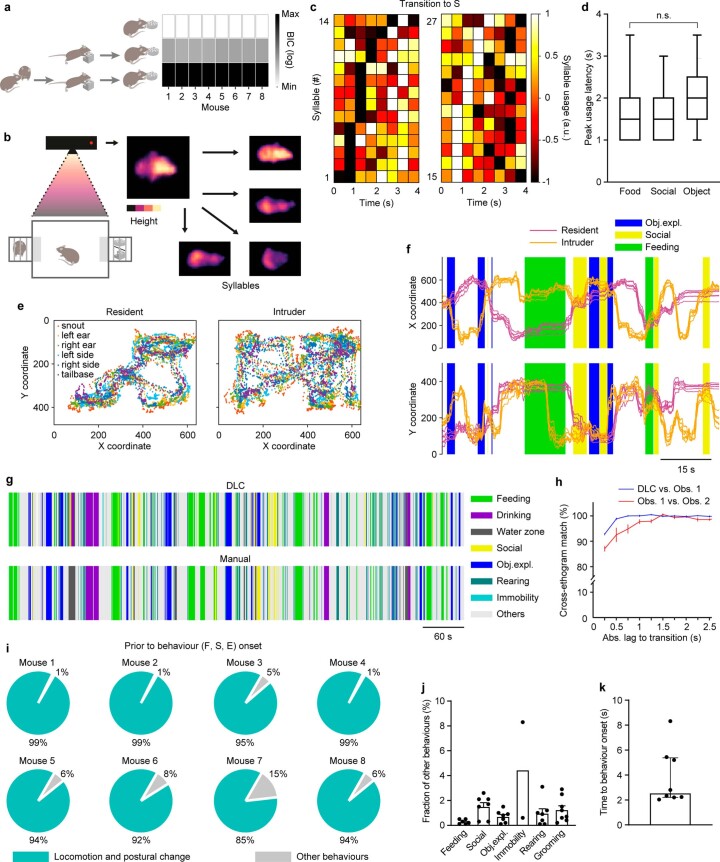
The organization of behavioral transitions. **a**, Markov chain models of all scored behaviors (pictograms, example sequences) featured the lowest Bayesian information criterion parameter for the model with order 0 (top row), N = 8 mice. **b**, Depth camera recordings in a free choice paradigm with food or a novel object and a conspecific behind a mesh. Mouse depth images were decomposed into syllables using MoSeq. **c**, Usage of syllables, sorted according to the time of the change in usage trends (peaks or troughs, whichever is closer to the transition point) over the period of 4 s before transitions to social contact. Colour bar: the probability in a set of 1000 shuffled epochs, normalized within and averaged across 9 mice. **d**, Average lag of syllables’ usage change (peaks and troughs, whichever is closer to the transition point) for transitions to feeding (n = 25 syllables), social contact (n = 29) and novel object exploration (n = 23, not different between syllables, p = 0.12, ANOVA), box outlines and center line, median and quartiles; whiskers, 1.5 x IQR. **e**, **f**, Markerless pose estimation using DeepLabCut (DLC). **e**, Trajectory of a resident (one of eight resident mice) and an intruder for an example epoch shown in **f**. **f**, Changes of the estimated position of the resident and the intruder during behaviors. **g**, Ethograms generated using DLC and manual behavioral scoring, representative mouse. **h**, CCGs of transition times, obtained by DLC-derived (reference) and manual scoring (Observer, Obs., 1) and between ethograms independently generated by different observers (Obs. 1, reference, vs. Obs. 2) in six mice. Cross-ethogram match: the difference between a CCG and a reference series ACG. **i**, **j**, Contribution of locomotion, unspecific postural changes vs. specific innate behaviors (**i**) and of individual behaviors (**j**, mean ± SEM) to combined transitions to feeding, social and novel object exploration, N = 8 mice. **k**, The duration of the dominant behavioral state preceding transition times – locomotion and unspecific postural changes, median ± IQR, N = 8 mice. See also Supplementary Information for the statistical information for **a**, **d**, **i**. Source data

To estimate the time frame of the information processing involved in behavioral transitions, we analyzed behavioral sequences using Markov chain models. The modeling revealed that accounting for preceding behaviors did not improve predictions about the upcoming one (Extended Data Fig. 2a). This suggested that behaviors (and transitions between them) are independent, and that neural dynamics driving transitions probably unfold close to the initiation of a new behavior. A specific time frame of transitions was further estimated using unsupervised motion segmentation (MoSeq^21^) in experiments separately testing each of the three behaviors (Extended Data Fig. 2b).

We reasoned that neural events driving transitions may be at least to some extent accompanied by concurrent changes of motion patterns. To reveal them, we analyzed the latency of the closest to the transition time stamps local minima or maxima in the occurrence of individual behavioral syllables. The occurrence of syllables was modified on average at 1.6, 1.7 and 2.0 s before food, social contact, and new object exploration, respectively (Extended Data Fig. 2c,d), suggesting that this might be a critical time window for a selection of a subsequent behavior. We next analyzed transitions in the free-choice task using manually scored ethograms. Their robustness and the precision of transition detection were validated by comparisons with an automatic scoring using markerless pose estimation^22^ and across observers (Extended Data Fig. 2e–h). In approximately 5% of transitions, F, S and E immediately followed one of the scored innate behaviors (Extended Data Fig. 2i,j). However, most times one of the three innate behaviors was preceded by locomotion and unspecific postural changes lasting approximately 2 s (Extended Data Fig. 2k). The subsequent neuronal analysis focused on 2-s epochs ending with the initiation of a new behavior, further referred to as transition epochs or transitions.

### Beta phase-dependent LH activity signals transitions

**Extended Data Fig. 3:**
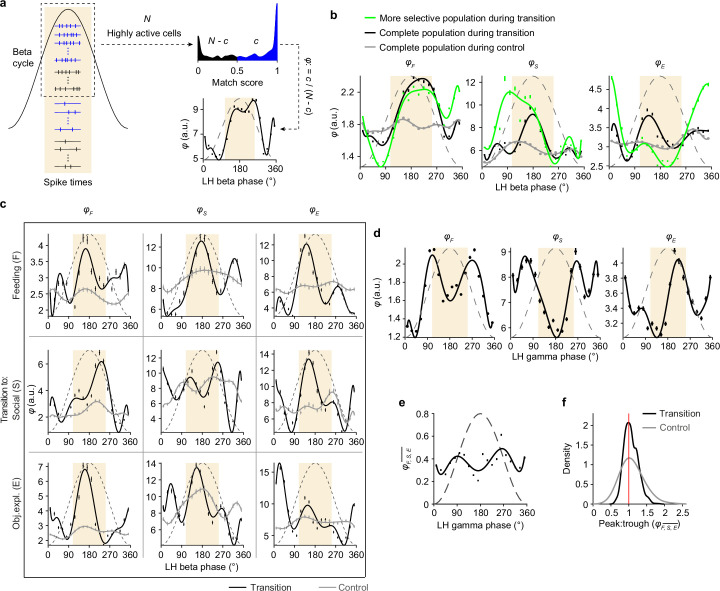
Phase signatures of LH neuronal discharge during transitions and behaviors. **a**, Definition of phase signatures for a population of LH cells. Ratios of high (*c*) to low match score (*N*-*c*) cells for each of the three behaviors are computed for sets of highly active cells, that is units with the firing probability in the upper quartile at each oscillation phase (binned). **b**, Beta phase signatures during transitions to feeding, social contact and novel object exploration (combined) and during 1000 shuffled sets of control epochs excluding these transitions for the complete LH population (see also normalized curves in Fig. 3a, n = 483 cells from six mice) and for a more selective LH population (match scores in lower or upper ten percentiles, n = 310 cells from six mice); Pearson correlation, phase signatures of complete vs. selective populations during transitions: *φ*_*F*_: r = 0.82, p < 0.0001, *φ*_*S*_: r = 0.58, p = 0.0071, *φ*_*E*_: r = 0.43, p = 0.0525; complete populations during transition vs. control: *φ*_*F*_: r = 0.25, p = 0.29, *φ*_*S*_: r = 0.29, p = 0.20, *φ*_*E*_: r = 0.02, p = 0.92. **c**, Beta phase signatures during transitions to behaviors and during 1000 shuffled sets using the same population of LH cells across behaviors, n = 88 cells from three mice. Columns: phase signatures for feeding (*φ*_*F*_), social contact (*φ*_*S*_) and novel object exploration (*φ*_*E*_). **d**, Gamma (30-60 Hz) phase signatures during transitions to feeding, social contact and novel object exploration (combined) and during 1000 shuffled sets (n = 439 cells from four mice). **e**, Average of gamma phase signatures shown in **d**. **f**, Peak:trough preference of the average gamma phase signature shown in **e**, during transition and 1000 sets of control epochs; transition, p = 0.42, control, p = 0.43, bootstrap test for peak:trough preference. Red line; phase-uniform firing distribution. Ivory shade, peak neighbourhood, four bins (72°) flanking the oscillation peak. Data are shown as mean ± 95% CI, bootstrap, polynomial fit. Dashed sine curve, reference cycle. See also Supplementary Information for the statistical information for **b**, **f**. Source data

To investigate phase coding of behavioral transitions, we considered that neuronal signaling during beta oscillations is largely determined by cells most active at a given oscillation phase. We studied behavior-related information signaled at a certain beta oscillation phase using a parameter referred to henceforth as a phase signature related to a particular behavior (Extended Data Fig. 3a). Phase signatures related to feeding (*φ*_F_), social contact (*φ*_S_) and new object exploration (*φ*_E_) were computed for the population of neurons most active at a given phase as the ratio of cells with match scores above and below 0.5, thus including all cells (Fig. 1f and Extended Data Fig. 3b); in a separate analysis, we considered only neurons with a high behavioral specificity (match scores above 0.9 or below 0.1; Extended Data Fig. 3b). Thus, a higher amplitude of the phase signature related, for instance, to feeding would indicate an increased output from feeding-related LH neurons at a certain beta oscillation phase. Surprisingly, phase signatures related to individual behaviors had consistently higher amplitudes near beta oscillation peaks (±72°, the peak neighborhood) during transition epochs compared to random nontransition control epochs of the same duration (Fig. 1f). This also held true for a population of neurons with high behavioral specificity (Extended Data Fig. 3b) or when phase signatures related to different behaviors were computed using the same subset of cells (Extended Data Fig. 3c), indicating the robustness against subsampling. In contrast, LH gamma oscillations (faster than 30 Hz), previously implicated in food-seeking^10^, did not feature the phase-coordinated signatures related to F, S and E during behavioral transitions (Extended Data Fig. 3d–f).

**Fig. 2:**
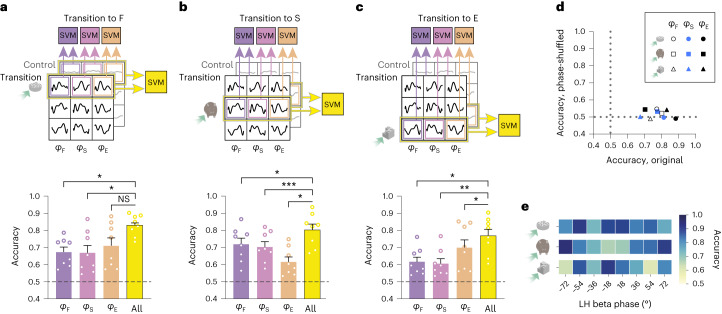
Encoding of behavioral transitions by beta phase signatures of LH neuronal populations. **a**–**c**, Schematics and bar charts showing the decoding of transitions to feeding (**a**), social contact (**b**) and new object exploration (**c**) versus control epochs using the phase signatures related to individual behaviors (top, violet, purple and peach) or their combination (right, yellow; ‘All’ bar) in SVMs computed separately for each phase bin in the peak neighborhood. Each data point represents the accuracy (mean of 1,000 cross-validations) in an individual phase bin; *n* = 110, 420 and 292 cells for the three types of transitions. *P < 0.0167, **P < 0.001, ***P < 0.0001, NS, not significant *P* = 0.02; adjusted *α* = 0.0167; paired *t*-test; the dashed line represents the chance level. Data are presented as the mean ± s.e.m. **d**, Accuracies of decoding transitions to different behaviors using original and phase-shuffled datasets. For each dataset, SVM-classified transition versus control epochs are shown. Each data point represents the accuracy for the original dataset (*x* axis) and the mean of accuracies across 1,000 phase-shuffled datasets (*y* axis). Randomization tests: *P* = 0.0009, 0.0019 and 0.0009, for transitions to F, S and E, respectively. The dotted line represents the chance level. **e**, Accuracies of decoding transitions to different behaviors versus control epochs using a combination of phase signatures related to individual behaviors in each SVM. Each element of the matrix represents the accuracy (mean of 1,000 cross-validations) in an individual phase bin, labeled according to the lag from the beta oscillation peak. See also Supplementary Information for the statistical information related to **a**–**e**. Source data

**Extended Data Fig. 4:**
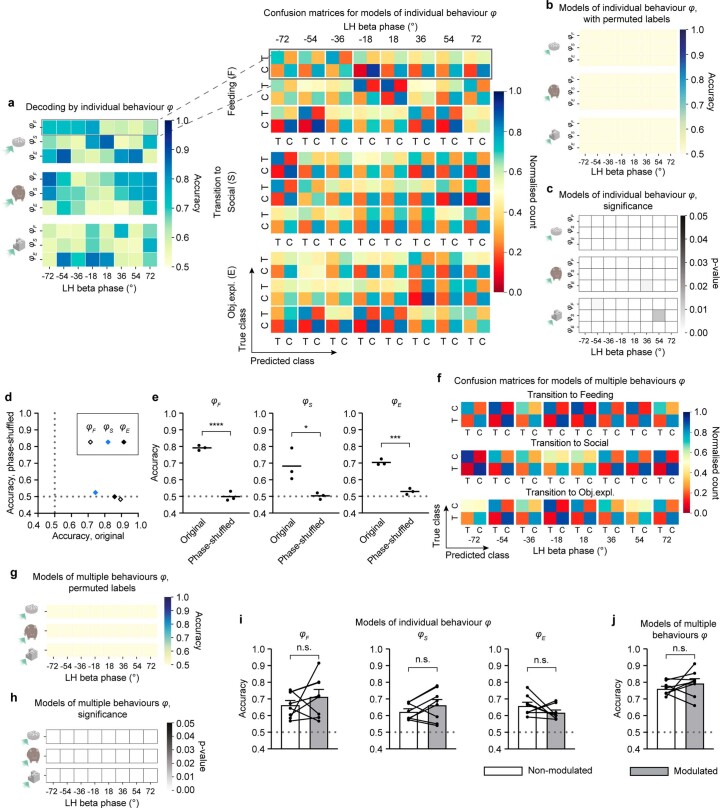
Decoding behavioral transitions by phase signatures of LH neuronal discharge. **a**, Left: Decoding transitions to different behaviors vs. control epochs by individual behavior phase signatures (populations of 110, 420 and 292 cells). Mean of n = 1000 cross-validations for each phase bin, with different lags from the beta oscillation peak. Right: confusion matrices. T: transition; C: control. **b**, Average decoding accuracies of models using individual behavior phase signatures after random reshuffling of class labels for each of the eight phase bins in the peak neighbourhood. **c**, Permutation test’s p-values for each model in (**b**). **d**, Decoding transitions to different behaviors (combined) using original and phase-shuffled datasets. SVMs classified transition vs. control epochs. Data points, accuracy for the original dataset (x-axis) and the mean accuracy across 1000 phase-shuffled datasets (y-axis), p = 0.0009, randomization test. **e**, Decoding transitions to different behaviors (combined) using original and phase-shuffled datasets of 197, 333 and 128 cells in individual mice. Data points, as in (**d**). *φ*_*F*_: p < 0.0001, *φ*_*S*_: 0.035, *φ*_*E*_: 0.0003, t-test. **f**, Confusion matrices of models shown in Fig. 2e, rows – transitions to different behaviors. T: transition; C: control. **g**, Average decoding accuracies of models using phase signatures of multiple behaviors derived otherwise as in (**b**). **h**, Permutation test’s p-values for models in (**g**). **i**, **j**, Decoding transitions to the three behaviors (combined) by phase signatures of 157 beta phase-locked vs. 514 non-modulated cells from six mice (mean ± SEM across eight bins in the peak neighbourhood) *φ*_*F*_: p = 0.33, *φ*_*S*_: 0.17, *φ*_*E*_: 0.23 and between models using phase signatures of multiple behaviors (‘All’), p = 0.32, t-test, paired between phase bins. Phase-locked cells: paired t-test, adjusted α = 0.0167, *φ*_*F*_ vs. All, p = 0.0465, *φ*_*S*_ vs. All, p = 0.0046, *φ*_*E*_ vs. All, p = 0.0007; non-modulated cells: *φ*_*F*_ vs. All, p = 0.0013, *φ*_*S*_ vs. All, p = 0.0005, *φ*_*E*_ vs. All, p = 0.0028. Dotted line, chance level. See also Supplementary Information for the statistical information for **d**, **e**, **i**, **j**. Source data

Next, we tested whether phase signatures related to individual behaviors could reliably differentiate between transition and control epochs. Support vector machine (SVM) classifiers were trained and tested separately in each phase bin in the peak neighborhood on 1,000 bootstrapped phase signatures from transition epochs versus the same number of phase signatures from time-shuffled control epochs. The decoding was performed using complete populations of highly active neurons as shown in Fig. 1f; as noted above, their phase signatures were similar to those of smaller subsets of highly behaviorally specific cells. Strikingly, transition to each behavior could be reliably decoded by individual phase signatures related to any of the three behaviors (Fig. 2a–c, statistical information in Supplementary Information and Extended Data Fig. 4a–c), suggesting that these phase-dependent neuronal populations signal a transition state. Importantly, the accuracy of decoding transition versus control epochs was markedly higher in the original than in the phase-shuffled data (Fig. 2d and Extended Data Fig. 4d,e).

To explore encoding of transitions by a combination of phase signatures related to individual behaviors, we next decoded the transition versus control epochs by including the phase signatures of all three behaviors in one model (Fig. 2a–c; ‘All’ SVMs). The approximate 80% accuracy of the models based on multiple behaviors exceeded the performance of almost all individual behavior-based models for the three types of behavioral transitions (Fig. 2a–c, Extended Data Fig. 4f–h and statistical information in Supplementary Information). In contrast to individual behavior-based models (Extended Data Fig. 4a, inset), the models using the phase signatures of all three behaviors predicted transitions more consistently across phase bins (Fig. 2e). Taken together, the LH population phase signatures related to different behaviors signaled transitions more reliably jointly than individually.

To address possible differences in the encoding of transitions depending on the entrainment of cells by beta oscillations, we compared the performance of SVMs (as in Fig. 2a–c), which included either modulated or nonmodulated neurons. Both populations signaled transitions with similar accuracies and also most accurately using a combination of phase signatures related to individual behaviors (Extended Data Fig. 4i,j), suggesting that the encoding of transitions is not limited to neuronal populations of cells with strongly beta-rhythmic responses.

### Phase signatures are better at encoding future than current behavior

**Fig. 3:**
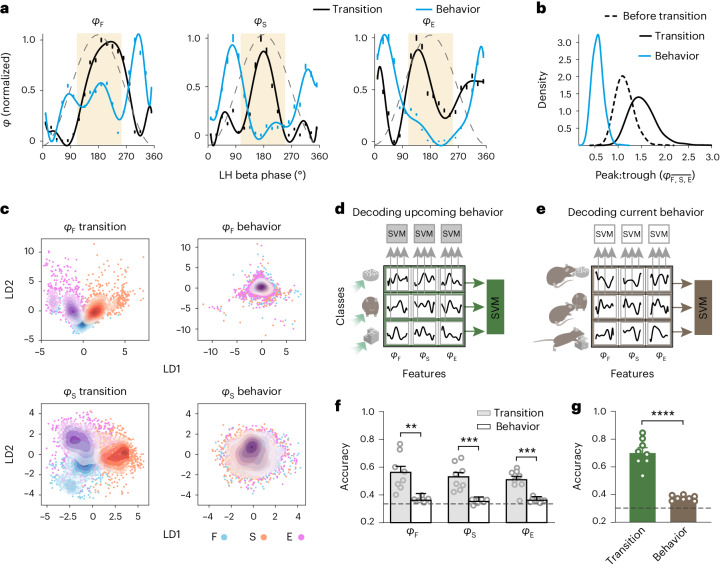
Beta phase signatures more accurately encode upcoming than current behaviors. **a**, Normalized phase signatures (bootstrap, polynomial fits) during combined transitions to F, S and E (*n* = 483 cells) and during the first 2 s of these behaviors (episodes lasting 8 s or longer, *n* = 205 cells from six mice). The ivory shade represents the peak neighborhood, that is, plus or minus four bins (72°). **b**, Peak:trough preference of average phase signatures computed for 2-s epochs before transitions (−4 s), during transitions and at behavior onset. Transition versus before transition and versus behavior; *P* < 0.0001; *t*-test. **c**, Phase signatures (first linear discriminants, LD1, LD2) related to feeding, *φ*_F_, and social contact, *φ*_S_, computed in the peak neighborhood during transitions to F (blue), S (orange) or E (purple dots) (left), and 2-s random epochs during these behaviors (right). The contours represent the probability density for each cluster. **d**, Decoding upcoming behaviors using phase signatures related to individual (top, gray) or multiple (right, green) behaviors in SVMs computed separately for each phase bin in the peak neighborhood during transitions (**f**,**g**, accuracies: transition). **e**, Decoding current behaviors using phase signatures related to individual (white) or multiple (brown) behaviors as in **d** but for 2-s random epochs during these behaviors (**f**,**g**, accuracies: behavior). **f**, Accuracies of decoding upcoming versus current behaviors using phase signatures related to individual behaviors using the models from **d** (gray) and **e** (white); *n* = 88 and 64 cells, respectively, from three mice; **P = 0.003, ***P = 0.0007, *α* = 0.0167; paired *t*-test. **g**, Accuracies of decoding upcoming versus current behaviors using a combination of phase signatures related to individual behaviors, using models from **d** (green) and **e** (brown); *n* = 88 and 64 cells, respectively, from three mice; ****P < 0.0001; paired *t*-test. Each data point in **f** and **g** represents accuracy (mean of 1,000 cross-validations) in an individual phase bin. Data are presented as the mean ± s.e.m. across eight bins in **a** as the mean ± 95% CI across the bootstraps. The dashed lines in **f**,**g** represent the chance level. See also Supplementary Information for the statistical information for **b**,**c**,**f**,**g**. Source data

**Extended Data Fig. 5:**
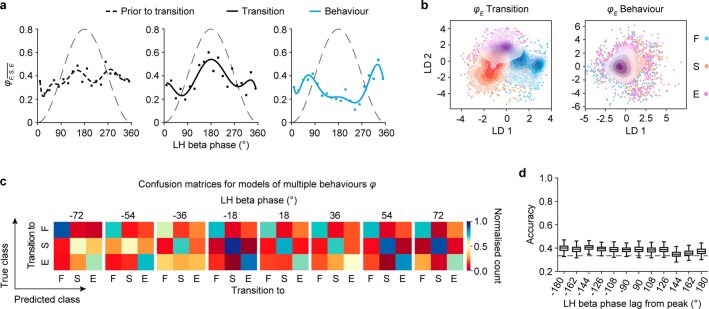
Decoding behavioral transition outcomes by phase signatures of LH neuronal discharge. **a**, Average of the phase signatures of feeding, social contact and novel object exploration computed for 2-s epochs prior to the transitions to these behaviors (left, −4 s, n = 472 cells), during transitions (middle, n = 483 cells) and upon behavior onset (right, n = 205 cells) from six mice, mean ± 95% CI, bootstrap, polynomial fit; dashed sine curve, reference cycle. **b**, Phase signature of novel object exploration, *φ*_*E*_ (represented by the first two linear discriminants, LD1, LD2), computed in the peak neighbourhood during transitions to F (purple dots), S (orange) or E (blue), left plots, and during 2-s random epochs during these behaviors, right plots. Contours represent the probability density estimated for each cluster. **c**, Confusion matrices of models shown in Fig. 3g (left bar, transitions). **d**, Accuracies of decoding the current behavior using phase signatures of multiple behaviors in each phase bin; box outlines and center line, median and quartiles of 1000 cross-validations; whiskers, 1.5x interquartile range; n = 64 cells, which fired during all the three behaviors, from three mice; dotted line, chance level. See also Supplementary Information for the statistical information for **b**. Source data

To study the encoding of ongoing behaviors by the collective neuronal activity in the LH during beta oscillations, we first compared phase signatures before, during and after transitions, that is, during the initial epochs of ensuing behaviors. Upon behavior onset, phase signatures sharply changed their pattern, becoming nearly antiphase with phase signatures during transitions (Fig. 3a and Extended Data Fig. 5a). Accordingly, the peak:trough ratio of a phase signature was greater than 1 during transitions compared to lower than 1 upon behavior onset and approximately 1, that is, phase-uniform, when preceding transitions (Fig. 3b).

We next examined whether the phase signatures of individual behaviors not only characterize behavioral transitions (Fig. 2a–c), but also predict the type of upcoming behaviors. To this end, we first performed linear discriminant analysis to evaluate the pattern of phase signatures during transitions to different behaviors and during those behaviors. Phase signatures related to an individual behavior substantially differed between transitions, depending on the type of the upcoming behavior (Fig. 3c and Extended Data Fig. 5b). In contrast, phase signatures were conspicuously similar during the three behaviors (Fig. 3c and Extended Data Fig. 5b). These observations were consistent with SVM classifications of phase signatures, which predicted upcoming F, S or E with a high accuracy of approximately 85% (statistical information in Supplementary Information for Fig. 3c and Extended Data Fig. 5b). Decoding of upcoming behaviors using phase signatures in individual beta phase bins was robust but less accurate than using all the bins near oscillation peaks (approximately 53%; chance level = 33.3%; Fig. 3f). In contrast, current behaviors were predicted with accuracies marginally above the chance level (Fig. 3e–g and Extended Data Fig. 5c,d). Furthermore, like the decoding of transitions versus control epochs (Fig. 2a–c), during transitions phase signatures related to different behaviors decoded upcoming behaviors approximately 21% more accurately jointly than individually (Fig. 3f,g). Conversely, decoding current behaviors using multiple phase signatures did not differ from decoding using phase signatures related to individual behaviors (Fig. 3f,g). Thus, the activity of behavior-related LH neuronal populations during beta oscillations more reliably predicted upcoming than current behaviors.

### Phase-specific LH activity is necessary for transitions

**Fig. 4:**
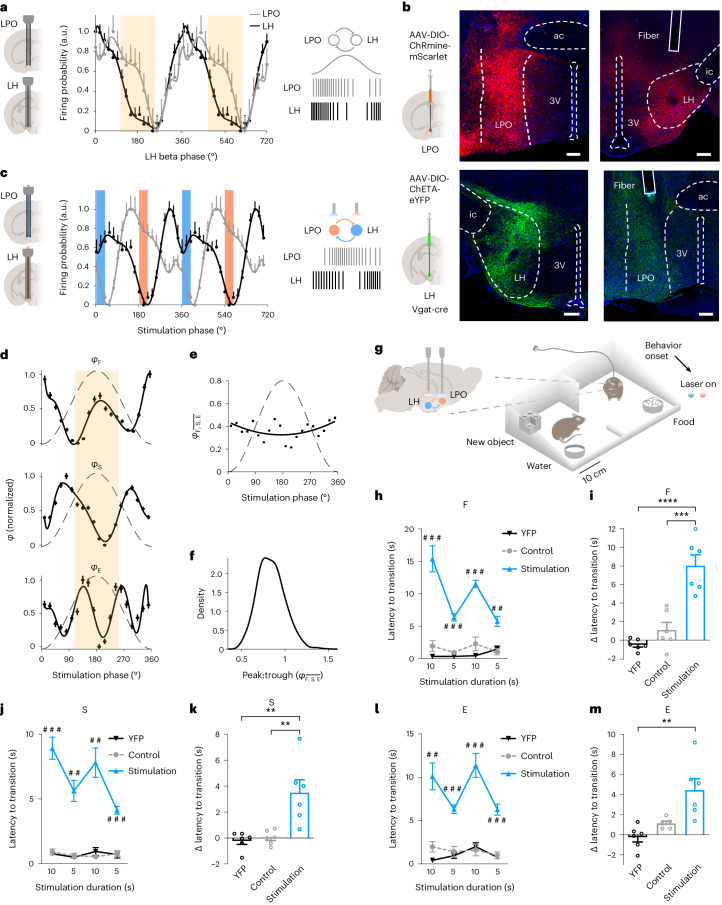
Behavioral transitions are suppressed by phase-specific intrahypothalamic inhibition. **a**, Firing probability (scaled from minimum to maximum) during beta oscillations (*n* = 1,871 LH cells from eight mice; *n* = 167 LPO cells from two mice); distributions were not significantly different; *P* = 0.1955; Mardia test. **b**, Opsin expression in the LH and LPO (one of six mice). 3V, third ventricle; ac, anterior commissure; ic, internal capsule. Scale bars, 200 μm. **c**, Firing probability of 52 LH and 21 LPO cells (*n* = 2 mice) inhibited by the stimulation of GABAergic LPO and LH afferents, respectively, during their out-of-phase beta-rhythmic stimulation (blue bar: 473-nm light; orange: 589-nm light); distributions are different; *P* < 0.0001; Mardia test. **d**–**f**, Normalized phase signatures for F, S and E (bootstrap, polynomial fit) computed during the out-of-phase stimulation (**d**), their average (**e**) and peak:trough preference (**f**); *n* = 193 LH cells; peak:trough preference not different from 1; *P* = 0.84; bootstrap test. The ivory shade represents the peak neighborhood. **g**, Optogenetic stimulation in the LPO and LH for 5 s or 10 s contingently upon the spontaneous onset of F, S or E (in separate experiments for each behavior). **h**–**m**, Beta-rhythmic out-of-phase stimulation eliminated the behavioral transitions. YFP, six mice expressing control constructs in LH (enhanced YFP (eYFP)) and LPO (mScarlet), beta out-of-phase stimulation; six mice expressing opsins: stimulation, beta out-of-phase; control, nonrhythmic stimulation with parameters matching the out-of-phase protocol; in-phase, beta in-phase stimulation in mice expressing opsins. **h**,**j**,**l**, Latency to transition during a 10-s or 5-s stimulation during F (**h**), S (**j**) or E (**l**); YFP versus stimulation; ^##^*P* < 0.001, ^###^*P* < 0.0001; unpaired *t*-test with *α* correction. **i**,**k**,**m**, Difference of latency to transition between a 10-s and 5-s stimulation during F (**i**), S (**k**) and E (**m**); **P < 0.01, ***P < 0.001, ****P < 0.0001; unpaired *t*-test with *α* correction. Data are presented as the mean ± s.e.m. In **d** data are presented as the mean ± 95% CI across bootstraps. See also Supplementary Information for the statistical information for **c**,**f**,**h**–**m**. Source data

To investigate the necessity of phase-specific activity in the LH for behavioral transitions, we used dual-site electrophysiological recordings to first characterize the timing of neuronal activity in the LH and its main intrahypothalamic inhibitory input, the LPO (Fig. 4a). GABAergic projections from the ventrolateral preoptic area to the caudal parts of the lateral zone of the hypothalamus were among the first identified intrahypothalamic circuits implicated in the regulation of innate behaviors, specifically in the regulation of the sleep–waking cycle^23^. More recent studies demonstrated the role of the preoptic area in general and the LPO in particular in social behaviors^5^. Prominent inhibitory connections between the LPO and LH make the recurrent LH–LPO circuit suitable for manipulating the timing of rhythmic activity in the LH. Neuronal activity in the LH and LPO was increased and coordinated during beta oscillations (Extended Data Fig. 1g). In detail, LPO neurons fired with a phase offset in relation to the maximal discharge in the LH; the firing probability of LPO cells remained elevated during peaks of beta oscillations (Fig. 4a and Extended Data Fig. 1h). Thus, in the reciprocally connected LH–LPO circuit, LH and LPO afferents were synchronously active in a phase-offset fashion.

**Extended Data Fig. 6:**
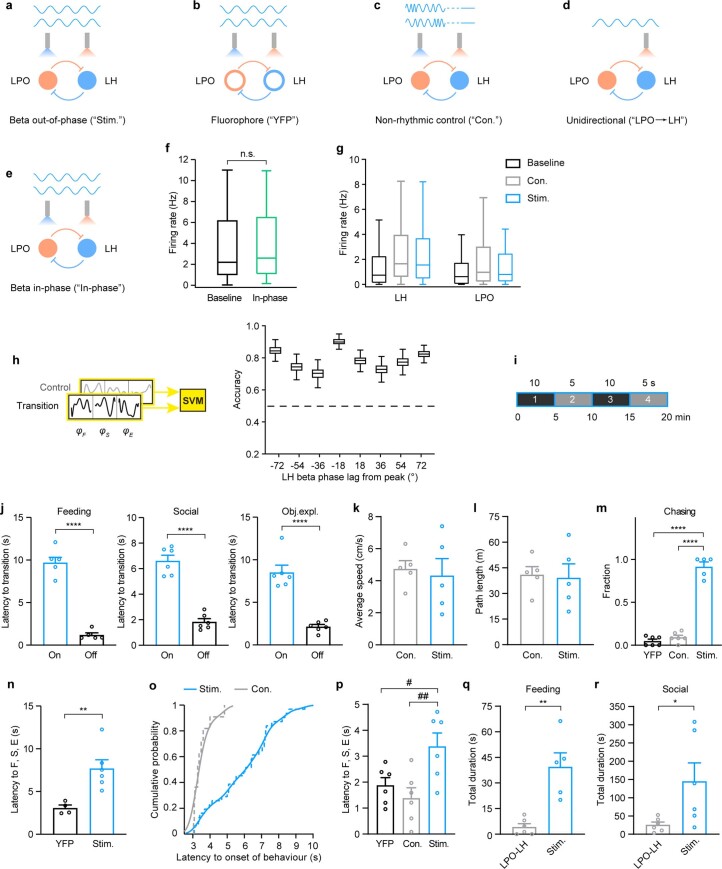
Causal role of LPO-LH beta oscillations in behavioral transitions. **a-e**, Beta out-of-phase stimulation in mice expressing opsins (**a**) and control constructs (**b**), non-rhythmic (**c**), a unidirectional LPO-to-LH (**d**) and in-phase stimulation (**e**). **f**, **g**, Firing rate during baseline vs. in-phase (**f**, n = 44 LH cells, 1 mouse, p = 0.054, signed-rank test) or vs. out-of-phase and non-rhythmic stimulation (**g**, n = 255 LH and 106 LPO cells, 2 mice, p = 0.4, ANOVA). **h**, Decoding of transitions from F, S and E (combined) to various behaviors vs. non-transition epochs; n = 423 cells, 3 mice; p < 0.0001, permutation tests. Dotted line, chance level. **i**, Stimulation for 10 and 5 s upon onset of F, S or E (in separate experiments). **j**, Latency to transition from F, N = 6 (On), N = 5 (Off); S and E, N = 6 mice, during stimulation (10, 5 sec light on epochs combined) upon behaviors and light off epochs. **k**, **l**, Average speed during out-of-phase and non-rhythmic (Con.) stimulation. N = 5, Con., Stim. mice, p = 0.7, t-test (**k**) and path length, 5 Con., Stim. mice, p = 0.9, t-test (**l**). **m**, Chasing (fraction of S episodes) during 10 s stimulation upon onset of S, N = 6 YFP, Con., N = 5, Stim. mice. **n**, Latency to F, S or E during out-of-phase and control (YFP) stimulation upon S. N = 4, YFP N = 5, Stim., p = 0.0076, t-test. **o**, Empirical CDF (with kernel estimate) of the latencies to F, S and E during out-of-phase and non-rhythmic (Con.) stimulation in the absence of these behaviors. N = 6 mice, Stim. vs Con., p = 0.0002, two-sample KS-test. **p**, Latency to F, S or E during stimulation in the absence of these behaviors. N as in (**o**). **q**, **r**, Duration of behavior during LPO-to-LH or out-of-phase non-contingent on behavior stimulation. **q**, F, N = 6, LPO-LH, 5, Stim. mice, p = 0.0014. **r**, S, N = 6, LPO-LH, Stim. mice, p = 0.042, t-test. F, Feeding, S, social contact, E, novel object exploration. Data in bar plots, mean ± SEM. Box outlines and center line, median and quartiles; whiskers, 1.5x IQR. *, p < 0.05, **, p < 0.01, ****, p < 0.0001, #, p = 0.03, ##, p = 0.01, t-test. See also Supplementary Information for the statistical information for **f**–**h**, **j**–**r**. Source data

To mimic and strengthen this temporal offset and thus enhance inhibition of LH cells during oscillation peaks, we entrained beta oscillations in the LH and LPO out of phase using a rhythmic optogenetic phase offset stimulation of inhibitory projections between both regions. For this purpose, we targeted a fast channelrhodopsin-2 (ChR2) variant ChETA to LH GABAergic cells and a red-shifted ChR2-variant ChRmine^24^ to LPO GABAergic cells in Vgat-cre mice (Fig. 4b). We then stimulated LH–LPO GABAergic projections at a beta frequency (20 Hz) out of phase with the stimulation of LPO–LH GABAergic projections (henceforth, out-of-phase stimulation; Fig. 4c and Extended Data Fig. 6a). This stimulation protocol offset the timing of inhibition to the LH by half a period of the optogenetically entrained LH beta oscillation (25 ms corresponding to 180°; Fig. 4c), that is, to the time of the transition-related activity in the LH (Fig. 1f). During the out-of-phase LH–LPO stimulation, the phase signatures of the three behaviors were uncoordinated, lacking the typical for behavioral transitions increase of their magnitude near oscillation peaks (Fig. 4d–f). As a control, in addition to mice that expressed a control construct without opsin (yellow fluorescent protein (YFP); Extended Data Fig. 6b), a nonrhythmic optogenetic stimulation (Extended Data Fig. 6c), unidirectional LPO–LH stimulation (Extended Data Fig. 6d) or in-phase stimulation at the beta frequency (Extended Data Fig. 6e) were applied. These manipulations of the LH neurons discharge timing were not accompanied by confounding changes of the average firing rates in the LH and LPO (Extended Data Fig. 6f,g).

To study the role of phase signatures near beta peaks in behavioral transitions, we applied out-of-phase stimulation during defined behaviors, prevalent in this task, that is, F, S or E, and quantified the latency to the next transition (that is, the duration of an ongoing behavior) compared to control stimulation protocols. This approach enabled the manipulation of the transition state in behaviorally defined conditions. Importantly, in spontaneous activity recordings during the final 2 s of the three behaviors, the phase signatures of LH neurons accurately predicted the upcoming transitions (Extended Data Fig. 6h). In separate experiments, optogenetic stimulation was applied in a free-choice model on a spontaneous onset of F, S or E (Fig. 4g and Extended Data Fig. 6a–e,i). The beta out-of-phase stimulation eliminated behavioral transitions, thereby markedly extending ongoing innate behaviors proportionally to the duration of the stimulation (5 or 10 s) compared to a nonrhythmic optogenetic stimulation in the same mice and to the stimulation in mice expressing a control construct (Fig. 4h–m, Supplementary Videos 1–3 and Extended Data Fig. 6j). Mice continued to engage in the behavior that they pursued immediately before the stimulation onset, with no changes in locomotion (Extended Data Fig. 6k,l). A striking example of persistent behaviors during out-of-phase but not control stimulation was the inability of mice to discontinue nonreciprocal social contact leading to chasing an escaping conspecific (Extended Data Fig. 6m and Supplementary Video 4). Similarly, transitions to the three behaviors, that is, F, S and E, were delayed by the out-of-phase stimulation (Extended Data Fig. 6n–p). Increased duration of feeding and social behaviors was also observed during the beta out-of-phase stimulation, which was not contingent on behaviors, compared to the unidirectional LPO–LH stimulation (Extended Data Fig. 6q,r), demonstrating the critical role of reciprocal intrahypothalamic inhibition in behavioral transitions.

**Extended Data Fig. 7:**
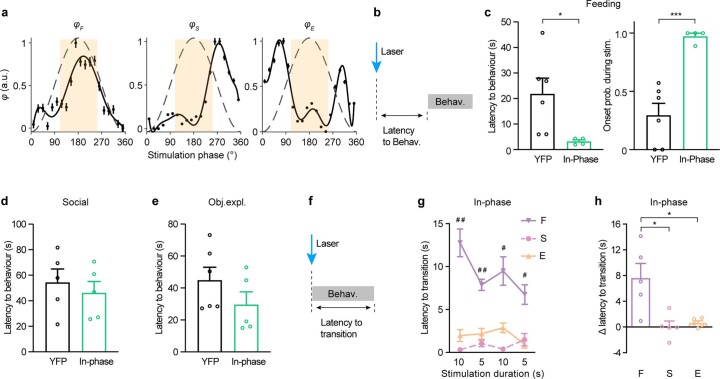
Facilitation of transitions to feeding by in-phase LPO-LH stimulation. **a**, Normalized phase signatures (bootstrap) for feeding, social contact and novel object exploration computed during 20-s epochs of the in-phase stimulation, n = 44 cells, mean ± 95% CI, polynomial fits, dashed sine curve, reference cycle; ivory shade, peak neighbourhood. Comparison of peak:trough preference of phase signature for each behavior vs. 1 - a uniform phase signature: feeding, p < 0.0001, social contact and novel object exploration, p = 1, one-sample t-test (right tailed). **b**, Schematic, measurement of the latency to feeding, social contact or novel object exploration (Behav.) after the onset of in-phase LPO-LH or control (mScarlet/YFP) stimulation (initiated upon social behavior). **c**, Latency to feeding and the probability of feeding onset during. N = 6 (YFP), 4 (In-phase) mice. YFP vs. In-phase, latency, p = 0.043; probability of onset, p = 0.0008, t-test. **d**, Latency to social contact. N = 5 (YFP), 5 (In-phase) mice. YFP vs. In-phase, latency, p = 0.6, t-test. **e**, Latency to novel object exploration. N = 6 (YFP), 5 (In-phase) mice. YFP vs. In-phase, latency, p = 0.2, t-test. **f**, Schematic, measurement of the latency to transition from feeding, social contact or novel object exploration (Behav.) after the onset of stimulation initiated upon these behaviors. **g**, Latency to transition during 10 s or 5 s in-phase LPO-LH stimulation during feeding (F), social contact (S) and novel object exploration (E). N = 5 mice. F vs. S, F vs. E, 0.0052 ≥ p ≥ 0.0002, F vs. S: p < 0.0001, t-tests, #, p < 0.01, ##, p < 0.001. **h**, The difference of latency to transition between 10 s and 5 s stimulation during feeding, social contact and novel object exploration. N = 5 mice. F vs. S: p = 0.015. F. vs. E: p = 0.017, t-tests. *, p < 0.05, ***, p < 0.001, t-test. Data are presented as mean ± SEM. See also Supplementary Information for the statistical information for **a**, **c**-**e**, **g**, **h**. Source data

To test whether the transition state could be further manipulated by a different phase relationship in the LH–LPO circuit, we computed the phase signatures of LH populations in relation to the three behaviors during in-phase LH–LPO stimulation. This stimulation was associated with a transition phase signature (near beta oscillations peaks) for feeding but not for social contact and new object exploration (Extended Data Fig. 7a). Accordingly, in-phase LH–LPO stimulation, initiated upon social contact, reduced the latency to feeding onset but not to new object exploration or new social contact episodes compared to the YFP group (Extended Data Fig. 7b–e). Furthermore, transitions from feeding were delayed by in-phase stimulation, in agreement with absent transition phase signatures for social contact and new object exploration during in-phase stimulation (Extended Data Fig. 7f–h). Together, these results indicate that manipulations of LH phase signatures affect behavioral transitions.

### LH ‘transition’ cells and mPFC coordination during transitions

**Fig. 5:**
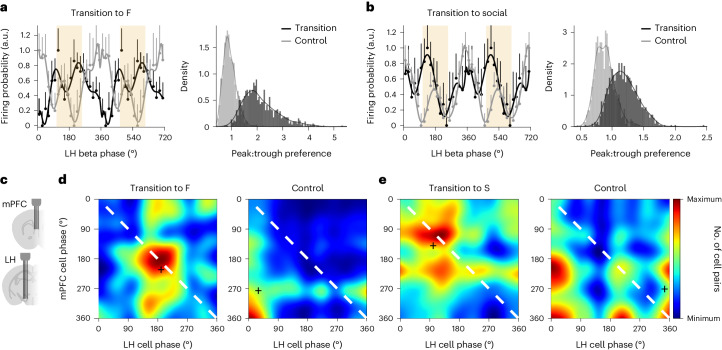
LH ‘transition’ cells are coordinated with mPFC neurons during behavioral transitions. **a**, Left: firing probability of LH multimodal cells (high match scores for all three behaviors) according to the LH beta oscillation phase during transition to feeding, and control epochs excluding transitions to any behaviors, scaled between zero and one; *n* = 59 cells from four mice; mean ± s.e.m.; polynomial fit. The ivory shade represents the peak neighborhood. Right: peak:trough preference of the discharge during transition versus control epochs; *P* < 0.0001; *t*-test. **b**, Left: firing probability of LH multimodal cells according to the oscillation phase during transition to social contact and control epochs; *n* = 141 cells from five mice; mean ± s.e.m.; polynomial fit. The ivory shade represents the peak neighborhood. Right: peak:trough preference of the discharge during transition versus control epochs; *P* < 0.0001; *t*-test. **c**, Schematic of the dual-site recordings in the LH and mPFC using movable silicon probes. **d**,**e**, Co-firing probability of simultaneously recorded LH and mPFC cells during LH beta oscillations. Histogram (normalized and convolved) showing the count of the LH and mPFC cell pairs with mean discharge phases in individual phase bins; high co-firing indicates increased count in the main diagonal (white dashed line); the black crosses indicate the mean phases of the bivariate distributions. **d**, Distributions from transitions to feeding versus control epochs; *n* = 351 cell pairs; *P* < 0.0001; bivariate likelihood ratio test. **e**, Transitions to social contact versus control epochs; *n* = 1,451 cell pairs; *P* < 0.0001. See also Supplementary Information for the statistical information for **a**,**b**,**d**,**e**. Source data

**Extended Data Fig. 8:**
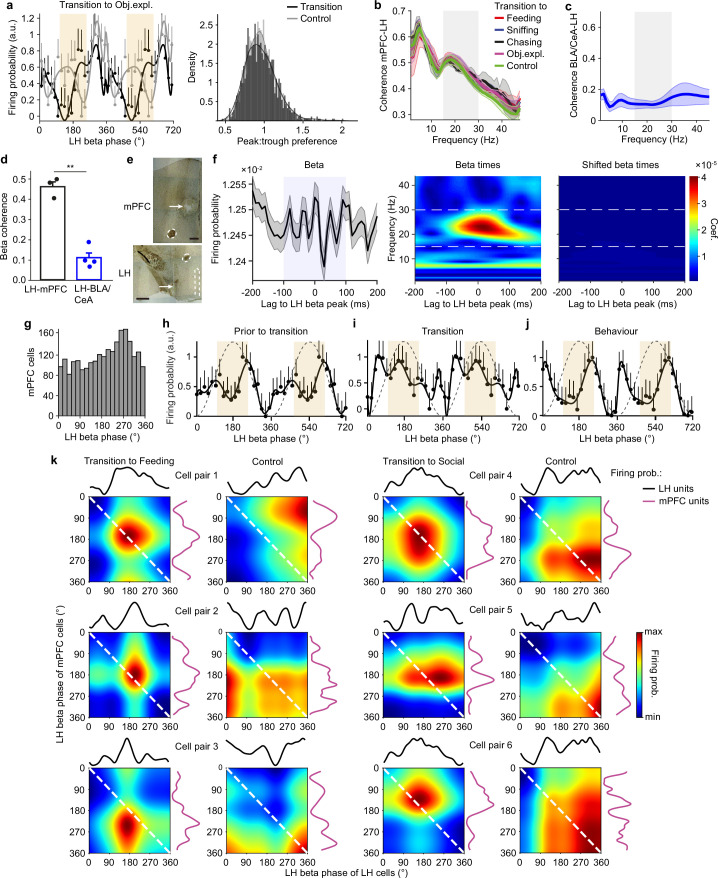
Beta-rhythmic mPFC-hypothalamic coordination during behavioral transitions. **a**, Left: firing probability of LH multimodal cells (scaled from 0 to 1) according to beta phase within 2 s prior to transition to E (n = 110 cells, 3 mice) and during control epochs in the whole recording sessions. Right: ratio of cumulative firing probabilities during peak phases to probabilities during trough phases during transition to E and control epochs, p = 0.59, t-test. **b**, Coherence of mPFC and LH LFP during transitions to innate behaviors and control epochs, N = 3 mice. **c**, Coherence of amygdala (BLA, basolateral, CeA, central nuclei) and LH LFP, complete recording sessions, N = 4 mice. **d**, Beta-band coherence, N = 3 (LH-mPFC), 4 (LH-BLA/CeA) mice, p = 0.0003, t-test. **e**, Dual-site recordings in mPFC and LH, brain sections showing silicon probes localization (arrows). Dashed lines, the anterior part of the anterior commissure (top) and the mammillothalamic tract (bottom). Scale bar, 200 μm. **f**, Probability of mPFC discharge in relation to the maximum amplitude of LH beta oscillation envelopes, n = 2374 cells, 3 mice (left), wavelet spectrograms of native (middle) and shuffled (right, shifting beta times by 10 sec) CCGs. **g**, Histogram of average LH beta phases for mPFC cells, n = 2374 cells, 3 mice. **h-j**, Firing probability of mPFC cells (scaled from 0 to 1) according to LH beta phase during 2-s epochs prior to transitions (−4 s) to the three combined behaviors (n = 552 cells) (**h**), during transitions (n = 556 cells) (**i**) and upon behavior onset (n = 334 cells) (**j**). N = 3 mice. **k**, Coherograms of six pairs of simultaneously recorded LH and mPFC cells. High co-firing corresponds to the diagonal of plots showing the joint firing probability of LH and mPFC cell pairs during transitions to F, shuffled transitions to F, transitions to S and shuffled transitions to S. Curves, firing probability distributions. Data are presented as mean ± SEM. Ivory shade, peak neighbourhood; fits, polynomial; grey shade (**b**, **c**), beta-band. See also Supplementary Information for the statistical information for **a**, **b**, **d**, **h**–**j**. Source data

A considerable population of LH cells increased their firing rate during each of the three studied innate behaviors (19% of LH cells). These multimodal cells strongly contributed to phase signatures during transition to feeding and social contact. While during transitions they were active close to the beta oscillation peaks, during the control epochs they fired close to the oscillation troughs (Fig. 5a,b). In contrast, during transitions to a new object exploration, feeding and social contact transition cells retained their preferential activity in troughs (Extended Data Fig. 8a).

To explore the cognitive regulation of behavioral transitions by inputs to the LH, we studied the coordination of the feeding and social contact transition cells in the LH with the activity in the mPFC, the main cortical input of the LH and a key region for the cognitive control of innate behaviors^10,25–28^. Simultaneously recorded LFP in the LH and mPFC, but not in the basolateral and central amygdala, were conspicuously coherent at beta frequencies (Extended Data Fig. 8b–e). This coordination was accompanied by a beta-rhythmic discharge of mPFC neurons during LH beta oscillations (Fig. 5c and Extended Data Fig. 8f) and by the entrainment of approximately 11% of individual mPFC cells to LH beta oscillations with a preferred phase of approximately 270° (see Extended Data Fig. 8g for all mPFC cells). As a population, mPFC cells fired according to the LH phase with several modes, both before transitions and during behavior (Extended Data Fig. 8h,j). During transitions (Extended Data Fig. 8i), firing of the mPFC population increased at multiple phases, including oscillation peaks, becoming distinct from pre-transition or behavioral epochs. Next, we analyzed the firing of simultaneously recorded pairs of mPFC and LH cells. LH ‘transition’ cells and mPFC cells fired in synchrony, close to LH beta oscillation peaks, selectively during transitions to feeding and social contact, but not during nontransition control epochs (Fig. 5d,e and Extended Data Fig. 8k).

### Beta-rhythmic mPFC inputs facilitate behavioral transitions

**Fig. 6:**
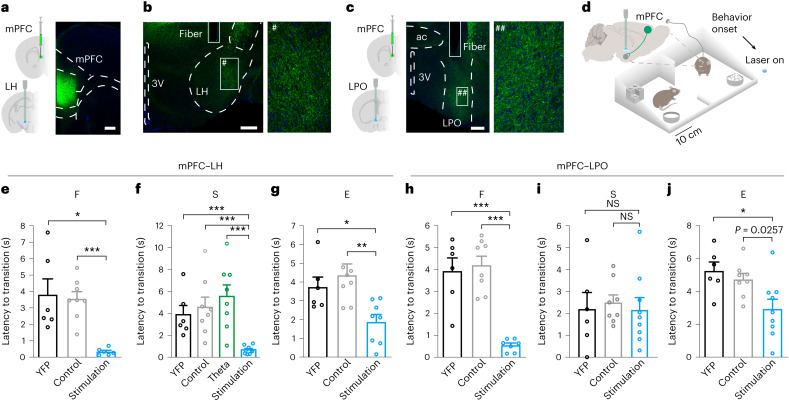
Beta-rhythmic mPFC–hypothalamic signaling promotes behavioral transitions. **a**–**c**, Optogenetic stimulation of mPFC–LH and mPFC–LPO projections. **a**, eNPAC2.0 expression in the mPFC (representative images; total *n* = 9 opsin mice). **b**,**c**, Left: optic fiber tracks above the LH and LPO. Right: magnification of a fragment # of **b** and ## of **c** (left). Scale bars, 200 μm (**a**), 250 μm (**b**,**c**). **d**, Beta-frequency stimulation of mPFC–hypothalamic projections on F, S or E. **e**–**g**, Optogenetic excitation of mPFC–LH projections reduced the latency to behavioral transitions. **e**, Feeding: blue light beta-frequency (20-Hz) stimulation, YFP, 20 Hz, six mice; control, eNPAC2.0, nonrhythmic stimulation with parameters matching the beta-frequency protocol, eight mice; stimulation, eNPAC2.0, 20 Hz, six mice. YFP versus stimulation, *P* = 0.0048; control versus stimulation, *P* < 0.0001; adjusted *α* = 0.025; unpaired *t*-test. **f**, Social contact: YFP, six mice; control, eight mice; theta, eNPAC2.0, theta frequency (9-Hz) stimulation, eight mice; stimulation, nine mice. YFP versus stimulation, *P* = 0.0004; control versus stimulation, *P* = 0.0006; theta versus stimulation, *P* = 0.0002; adjusted *α* = 0.0167; unpaired *t*-test; **g**, New object exploration: YFP, six mice; control, eight mice; stimulation, eight mice. YFP versus stimulation, *P* = 0.015; control versus stimulation, *P* = 0.0042; adjusted *α* = 0.025; unpaired *t*-test. **h**–**j**, Effects of optogenetic excitation of mPFC–LPO projections on the latency to behavioral transitions. **h**, Feeding: YFP, six mice; control, eight mice; stimulation, seven mice; YFP versus stimulation, *P* < 0.0001; control versus stimulation, *P* < 0.0001; adjusted *α* = 0.025; unpaired *t*-test. **i**, Social contact: YFP, six mice; control, eight mice; stimulation, nine mice; YFP versus stimulation, *P* = 0.98; control versus stimulation, *P* = 0.64; adjusted *α* = 0.025; unpaired *t*-test. **j**, New object exploration: YFP, six mice; control, eight mice; stimulation, nine mice; YFP versus stimulation, *P* = 0.018; control versus stimulation, *P* = 0.026; adjusted *α* = 0.025; unpaired *t*-test. Data are presented as the mean ± s.e.m. *P < α, **P < 0.01, ***P < 0.001. See also Supplementary Information for the statistical information for **e**–**j**. Source data

**Extended Data Fig. 9:**
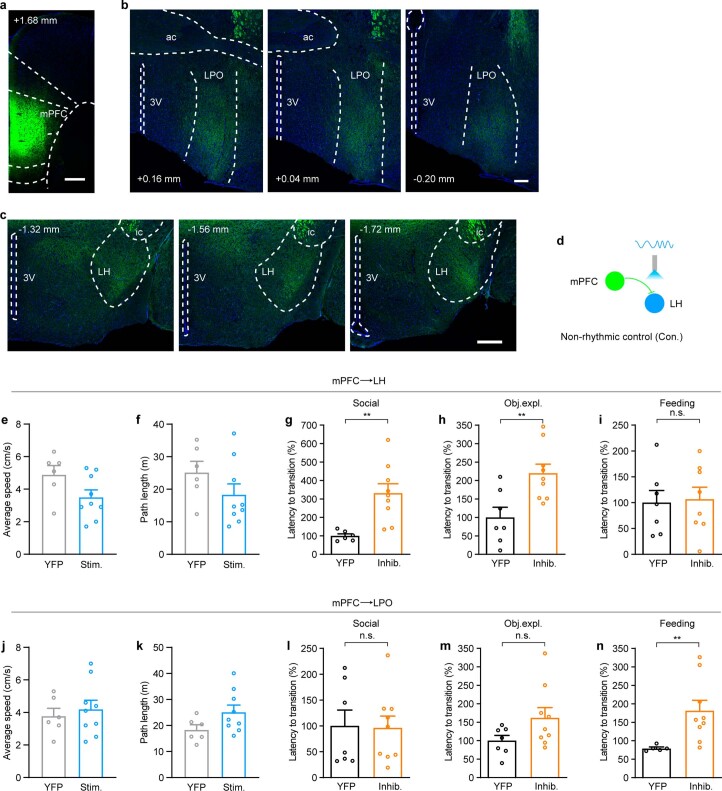
Causal role of the beta-rhythmic mPFC-hypothalamic signalling in behavioral transitions. **a**, Representative image showing eNPAC2.0 expression in the mPFC. Section coordinates: bregma +1.68 mm; scale bar, 200 μm (repeated in nine opsin mice). **b**, **c**, Representative images showing the projections of mPFC neurons in the LPO (**b**) and LH (**c**). Numbers on images - section coordinates, bregma. 3 V, third ventricle, ac, anterior commissure, ic, internal capsule. Scale bar, 150 μm (**b**), 250 μm (**c**) (repeated in nine opsin mice). **d**, Schematic of a non-rhythmic optogenetic stimulation (with light irradiation matched to the beta-rhythmic stimulation) of the mPFC-LH pathway. **e**, **f**, Locomotor activity during optogenetic excitation of mPFC-LH. **e**, Average speed, 6 (YFP), 9 (Stim.) mice, p = 0.07, t-test. **f**, Path length, 6 (YFP), 9 (Stim.) mice, p = 0.2, t-test. **g-i**, Effects of optogenetic inhibition of mPFC-LH projections on the latency to behavioral transitions (normalized to YFP). **g**, Social contact, N = 6 (YFP), 9 (Inhib.) mice, p = 0.003, t-test. **h**, Novel object exploration, N = 7 (YFP), 9 (Inhib.) mice, p = 0.006, t-test. **i**, Feeding, N = 7 (YFP), 8 (Inhib.) mice, p = 0.9, t-test. **j**, **k**, Locomotor activity during optogenetic excitation of mPFC-LPO projections. **j**, Average speed, 9 (Con.), 9 (Stim.) mice, p = 0.6, t-test. **k**, Path length: 6 (Con.), 9 (Stim.) mice, p = 0.08, t-test. **l-n**, Effects of optogenetic inhibition of mPFC-LPO projections on the latency to behavioral transitions (normalized to YFP). **l**, Social contact, N = 7 (YFP), 9 (Inhib.) mice, p = 0.9, t-test. **m**, Novel object exploration, N = 7 (YFP), 9 (Inhib.) mice, p = 0.09, t-test. **n**, Feeding, N = 5 (YFP), 9 (Inhib.) mice, p = 0.002, Mann-Whitney test. Data presented as mean ± SEM, *, p < 0.05, **, p < 0.01. See also Supplementary Information for the statistical information for **e**–**n**. Source data

To address the causal role of mPFC–LH projections in transitions between innate behaviors, we targeted AAVdj-hSyn-NpHR-TS-p2A-hChR2(H134R)-eYFP (eNPAC2.0), an opsin variant for opposing control of neuronal excitability^10^, to mPFC neurons and stimulated or inhibited their projections in the LH or LPO, or delivered light of the same wavelengths and patterns in YFP controls (Fig. 6a–d and Extended Data Fig. 9a–c). To mimic a more prominent input from the mPFC across phases, observed during behavioral transition (Extended Data Fig. 8i), mPFC–LH projections were stimulated at a beta frequency (20 Hz) without a specific phase relationship to LH oscillations. The stimulation applied in the same behavior-contingent fashion as LH–LPO stimulation during F, S or E, in separate experiments, shortened the latency to behavioral transitions (Fig. 6e–g and Supplementary Videos 5 and 6). In contrast, a nonrhythmic (Extended Data Fig. 9d) or a theta frequency (9-Hz) stimulation applied during social contact did not influence the latency to transition (Fig. 6f). Stimulation of mPFC–LH projections at a beta frequency (20 Hz) did not affect locomotion (Extended Data Fig. 9e,f). The inhibition of mPFC projections to the LH increased the latency of behavioral transitions for social contact (Extended Data Fig. 9g) and new object exploration (Extended Data Fig. 9h), but not for feeding (Extended Data Fig. 9i). Together, these results suggest that social contact and new object exploration are regulated by the mPFC–LH pathway, while food intake can be terminated by mPFC signals to the LH and by further pathways.

Considering the close LH–LPO interactions and the innervation of the LPO by the mPFC, we explored the behavioral functions of the mPFC projections to the LPO. The beta frequency stimulation of the mPFC–LPO projections did not affect the latency of the transitions during social contact, but it facilitated transitions during feeding and new object exploration (Fig. 6h–j). The average running speed and path length did not change upon stimulation (Extended Data Fig. 9j,k). Conversely to the effect of PFC–LH pathway inhibition, inhibition of the mPFC–LPO projections during social contact and new object exploration did not affect latency to transition (Extended Data Fig. 9l,m), whereas increased latency of transitions was observed for feeding behavior (Extended Data Fig. 9n). Thus, during F, S and E, prefrontal projections to the LH and LPO have complementary behavior-specific roles in transitions and together support their whole spectrum.

### Beta oscillations organize hypothalamic output to the VTA

**Fig. 7:**
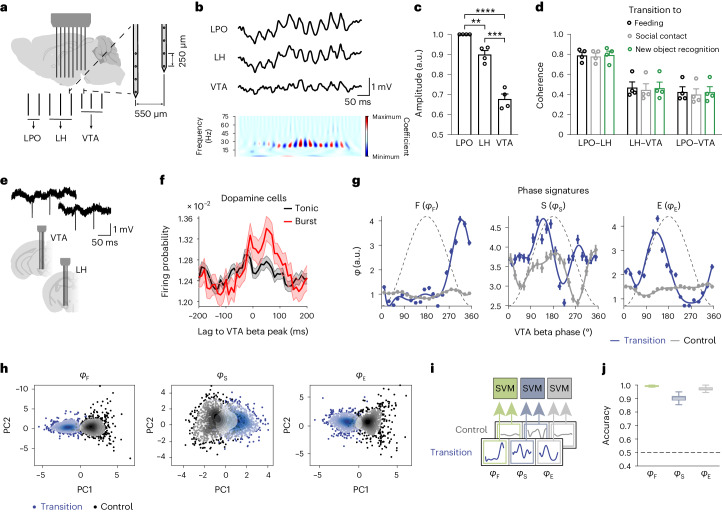
Lateral hypothalamic–VTA beta oscillations regulate the firing of dopaminergic neurons. **a**, Simultaneous recordings of LFP in the LPO, LH and VTA. **b**, LFP signal traces showing coordinated beta oscillations (1–100 Hz band pass) in the LPO, LH and VTA during social contact, and spectrogram of the VTA trace. **c**, Beta oscillation amplitude during behavioral transitions, normalized to the amplitude in the LPO; *n* = 4 mice; LPO versus LH, *P* = 0.002; LPO versus VTA, *P* < 0.0001; LH versus VTA, *P* = 0.0004. **P < 0.01, ***P < 0.001, ****P < 0.0001; adjusted *α* = 0.0167; unpaired *t*-test. **d**, Beta oscillation coherence during transitions to F, S and E; *n* = 4 mice; ‘behavior’, *P* = 0.9; analysis of variance (ANOVA). **e**, Top: burst discharge of a presumed VTA dopamine neuron during beta oscillations (1 Hz–10 kHz band pass signals). Bottom: dual-site VTA and LH recordings using movable silicon probes. **f**, Probability of tonic and burst discharge (interspike intervals greater than 170 and smaller than 80 ms, respectively) of putative dopamine neurons in relation to the maximum amplitude of VTA beta envelopes; *n* = 308 cells from three mice; tonic versus burst firing probability, *P* < 0.0001; paired *t*-test. **g**, Phase signatures of putative dopamine neurons during combined transitions to F, S and E and during 1,000 shuffled sets of control epochs excluding these transitions; mean ± 95% CI; *n* = 40 cells; polynomial fits. **h**, Phase signatures for F, S and E shown in **g** visualized according to their first two principal components. The contours represent the probability density estimated for each cluster. **i**, Decoding transitions to F, S or E (combined) versus control epochs using VTA^dopamine^ population phase signatures in all phase bins related to individual behaviors. **j**, Decoding transitions versus control epochs by VTA^dopamine^ cells during beta oscillations; box outline, center line, median and quartiles of 1,000 cross-validations; the whiskers represent 1.5 × the interquartile range; *P* < 0.0001; permutation tests of classifications; the dashed line represents the chance level. See also Supplementary Information for the statistical information for **c**,**d**,**f**,**g**,**j**. Source data

**Extended Data Fig. 10:**
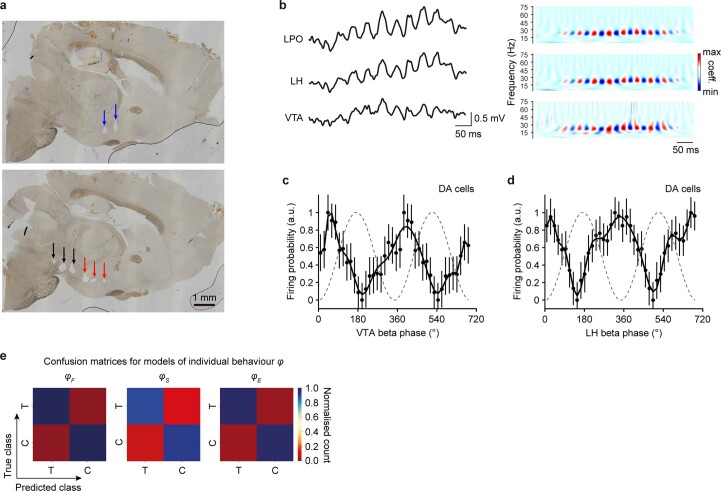
Beta oscillations and encoding of behavioral transitions in the VTA. **a**, Representative brain sections showing a silicon probe track in the VTA (black arrows, bottom section), LH (red arrows, bottom section) and LPO (blue arrows, upper section). **b**, Representative LFP traces showing simultaneously recorded beta oscillations during locomotion in the LPO, LH and VTA (1-100 Hz band-pass) and their respective spectrograms (wavelet scalograms). **c**, Firing probability of presumed dopamine cells according to the phase of VTA beta oscillation, n = 296 cells from three mice. **d**, Firing probability of presumed dopamine cells according to the phase of LH beta oscillations, n = 150 cells from two mice. **e**, Confusion matrices of models shown in Fig. 7j. Data presented as mean ± SEM. DA cells, dopamine cells. Source data

Next, we investigated the coordination of the hypothalamic transition dynamics with the neuronal activity in the VTA, the main lateral hypothalamic output region integral to multiple vital motivated behaviors. Beta oscillations were identified in approximately 22% of the recorded VTA signal. Multisite LFP recordings from the LPO, LH and VTA revealed oscillations of the highest amplitude in the LPO, closely followed by the LH and of approximately 30% lower amplitude in the VTA (Fig. 7a–c and Extended Data Fig. 10a,b). Beta oscillations were markedly coherent across regions (Fig. 7d) and modulated the discharge of putative dopaminergic neurons (Fig. 7e and Extended Data Fig. 10c,d). During beta oscillations, burst discharge of dopamine neurons (interspike intervals shorter than 80 ms (ref. ^29^) was more prominently elevated than tonic firing (Fig. 7f).

Like the phase signatures of LH neurons, the phase signatures of dopamine neurons during transitions to social contact and new object exploration featured an elevated amplitude close to the beta oscillation peaks (Fig. 7g). In striking contrast to our findings in the LH, in the VTA, the highest amplitude of phase signatures related to feeding was found in the oscillation troughs (Fig. 7g). A visualization of the phase signatures for all phase bins revealed their distinct features during transitions compared to control behavioral epochs (Fig. 7h). To validate these observations, we trained decoders to classify transition versus control epochs using the behavioral signatures of dopamine neurons at all phases in the same model, to account for their possible phase offsets in relation to the LH (Fig. 7i). High decoding accuracies for each phase signature were obtained (Fig. 7j and Extended Data Fig. 10e). Taken together, VTA dopamine neurons showed an increased activity during beta oscillations when their behavior-specific populations exhibited the periodic activity either in phase or out of phase with LH populations and predicted behavioral transitions.

## Discussion

In this work, we combined neuronal and LFP recordings in a free-choice model across the mPFC → (LPO↔LH) → VTA circuit, correlative and neural decoding approaches and optogenetics to explore the neuronal dynamics underpinning transitions between innate behaviors. We revealed a dynamic state preceding behavioral transitions, which is characterized in the LH by signaling of possible upcoming behaviors close to the peaks of previously unknown hypothalamic and VTA beta oscillations. The transition state accurately predicted the timing and order of innate behaviors and was necessary for transitions between them. We found that during transitions, phase-dependent population coding in the hypothalamus is coordinated with the beta-rhythmic firing in the mPFC, projections of which to the LH and LPO jointly regulate F, S and E. The transition state involves the VTA, where dopamine neurons increase firing and collectively signal transitions during beta oscillations.

Cortical beta oscillations have been implicated in sensory processing, movement control and short-term memory^18,19^, and involve inhibitory interneurons, including fast spiking and low-threshold cells, excitatory cells, communication between cortical layers, gap junctions and cholinergic modulation^19,30^. While the role of these mechanisms in oscillatory properties of the hypothalamic and VTA networks remains to be established, entrainment of the neuronal discharge indicates that beta oscillations coordinate the timing of neuronal activity within and across the regions studied in this work, that is, the LH, LPO, VTA and mPFC.

The finding of the phase-coordinated population signatures agrees with earlier computational modeling predicting robust temporal coding supported by feedback inhibition during beta oscillations^19^. In contrast to cell ensembles associated with gamma oscillations^31^, whose stability is strongly influenced by competing inputs^32^, beta oscillations in the cortex maintain sensorimotor and cognitive states^20^. The information processing advantages of beta oscillations, as demonstrated by modeling studies, include the resistance of phase-specific neuronal populations to fluctuations in excitability caused by external inputs. In turn, this results in the firing of additional spikes within cell ensembles without destabilizing them, thereby allowing new computations^19^. Transition phase signatures could be eliminated using a phase-specific recruitment of inhibition to the LH via LH^VGAT^–LPO^VGAT^ out-of-phase stimulation or partially mimicked by in-phase stimulation. Recurrent in-phase LH^VGAT^–LPO^VGAT^ stimulation most probably influenced feeding-inducing LH^VGAT^ cells^1,10^ with greater temporal precision than other, non-VGAT, LH cells involved in social contact and exploration (for example, orexin (hypocretin) neurons^33^), and thus produced a feeding-related LH transition phase signature.

Different innate behaviors evoked by electrical stimulation of the LH^34^ have previously been linked to the activation of partially overlapping cell populations involved in feeding, sleep, social and exploratory behavior, and appetitive motivation^1,6,34^. The present results indicate that cells with strongly conjunctive firing profiles, referred to in this study as ‘transition’ cells to emphasize their phase-shifted firing during transitions, coalesce with less conjunctive and with single behavior-preferring LH cells, forming phase-dependent representations of alternative upcoming behaviors, allowing their selection during transitions. Further research is required to elucidate possible transformations of phase-specific patterns into firing rate changes known to generate and maintain behavioral output^1,3–6^.

Learned behaviors, the execution of which is controlled by the mPFC, often include innate patterns prioritized by the hypothalamus. The importance of functional interactions between the mPFC and hypothalamus is further underscored by prominent projections of mPFC areas to the LH and by the involvement of the mPFC in feeding, social and novelty-driven behaviors^35^. By processing different sets (visceral-metabolic and sensory-mnemonic) of signals, both regions have a central role in providing an adaptive bias during behavior selection^28,36^. The present results suggest that the underlying transition dynamics entails the influence of prefrontal afferents on the LH. Conjunctive ‘transition’ LH cells are coordinated with mPFC inputs, which signal multidimensional population representations of adaptive behavior^37^. The representations of behavioral decision variables^38^, including the encoding of feeding and social interaction, by individual neurons in another frontal cortical LH input region, the orbitofrontal cortex, may influence innate behavior-selective LH cells during transitions.

Motivations driving innate behaviors are generated in the hypothalamus^34^. Both LPO and LH GABA neurons send direct projections to the VTA^5,6,39^ and influence the activity of VTA dopaminergic neurons^8^, which encode reward contingencies^40^ and diverse sensory, motor and cognitive variables^41^, and are indispensable for vital motivated behaviors^42^. Optogenetic activation of the LPO, and of LH^GABA^–VTA projections, promotes appetitive behaviors^43,44^, including sucrose seeking^6^, social interaction and new object exploration^45^, suggesting involvement of these pathways in the regulation of multiple innate behaviors. Activation of LH^GABA^ projections to the VTA at beta frequencies induced both feeding and intracranial self-stimulation, in contrast to stimulation at lower or higher frequencies, which either induced feeding or self-stimulation, respectively^44^.

Our results extend the repertoire of network rhythms organizing neuronal activity in the VTA^46^ and indicate that oscillations coordinate the communication from the hypothalamus to the VTA during epochs of an increased discharge of dopamine neurons. While the phase signatures of innate behaviors were coordinated between the LH and VTA via coherent beta oscillations, they were distinct between these regions. In contrast to the LH, the phase signatures of VTA cells for feeding and social contact versus new object exploration were observed approximately 25 ms apart (out of phase in the 20-Hz cycle). This delay could be due to the distinct properties (GABAergic versus glutamatergic cells) and connectivity (targeting dopaminergic versus GABAergic VTA cells) of the LH cells that are preferentially involved in one of the three behaviors. Dopamine neurons are also a heterogenous population, the activity of which is highly dependent on behavior, predicting future rewards and specific behavioral variables, such as position, velocity, previous reward and response accuracy, as well as behavioral outcomes^40,41,47,48^ and representing internal needs^49^. The present results suggest that during beta oscillations, the elevated and temporally separate activity of dopaminergic cells with distinct behavioral profiles differentially modulates efferent populations, thereby potentially contributing to the impact of lateral hypothalamic motivational signals on the planning and selection of action by the ventral striatum and prefrontal cortex^50^. Future studies will evaluate the influence of temporally coordinated lateral hypothalamic signals on VTA cells with distinct connectivity and electrophysiological properties.

Classical studies demonstrated preparatory activity in the supplementary motor cortex, which precede by less than a second not only specific self-initiated actions (by approximately 0.8 s) but also awareness about them (by approximately 0.5 s)^51,52^. In this study, we showed that a preparatory transition state emerges in the LH approximately 2 s before behavior onset as an oscillatory neuronal dynamics, which enables transitions between innate behaviors and encodes their selection.

Innate behaviors aiming at achieving basic needs, feeding and social interaction, are tightly interconnected in health and pathology. Both humans and rodents consume more food in the presence of conspecifics and adopt food preferences from conspecifics^53^; food consumption is linked to social hierarchy^54^ and hunger reduces the pursuit of mating opportunities^55^. Neural circuits regulating feeding and social behaviors partly overlap^56^. Furthermore, multiple neuropsychiatric disorders involve changes in both behaviors: for example, eating disorders are frequently comorbid with social phobia^57^, whereas autism spectrum disorders are often accompanied by disrupted eating behaviors^58^. Responses to novelty are also tightly linked to feeding and to social interactions: exposure to a new environment suppresses feeding behavior in mice^59^; animals spend more time with a new conspecific than with a familiar one^60^, suggesting overlapping circuits encoding those behaviors. All the three behaviors are goal-directed and can be cognitively regulated. In this study, we demonstrated that inputs from the PFC to the LH and LPO regulate transitions in a behavior-specific, complementary way, thus supporting the whole spectrum of transitions from those behaviors. Beta-rhythmic mPFC–LH signaling prompted transitions during all three studied behaviors. Inhibition experiments suggested that the activity of this pathway is necessary for timely transitions during social contact and new object exploration, while termination of feeding in the free-choice model relies on the beta-rhythmic activity of the mPFC–LPO projections. Thus, the mPFC–LPO pathway supports cognitively controlled cessation of food intake, which is also regulated depending on external and metabolic stimuli by other circuits, including those entailing the LH^61,62^. Further studies investigating other innate behaviors, for example, innate fear or aggression, would shed light on joint and distinct neuronal mechanisms of other behavioral transitions.

Collectively, our findings reveal a hypothalamic neuronal coding that underlies behavioral transitions. This dynamic transition state is supported by beta oscillations, which synchronize multiple hypothalamic behavioral codes. The hypothalamic transition state is coordinated with the VTA, the main output of the hypothalamus that is crucial for motivation and reward, and with the PFC, which promotes behavioral transitions. Disruptions of the transition state may contribute to diverse treatment-resistant behavioral dysfunctions common to psychiatric disorders, including eating disorders, and maladaptive social behaviors.

## Methods

### Animals

All animal procedures were performed in accordance with national and international guidelines and were approved by the local health authority (Das Landesamt für Natur, Umwelt und Verbraucherschutz). For this study, 10–25-week-old Vgat-ires-cre knock-in mice (The Jackson Laboratory) and C57BL/6 male mice were used, except for studies using MoSeq, which involved female mice. Mice were housed under standard conditions (air temperature 20–24 °C, relative humidity 45–65%) in the animal facility and kept on a 12 h light–dark cycle. Before all experiments, mice were handled by the experimenter and habituated to the experimental enclosure for 3–5 days^63^. This habituation procedure is important for minimizing the potential influence of unfamiliar experimental procedures or enclosure’s novelty on innate behaviors, for example, to ensure that animals consume food pellets in the experimental enclosure. Before the experiments with the optogenetic manipulations contingent on feeding, food was taken out from home cages for about 1 h; mice received water ad libitum.

### Viral injections

Viral injections in the LH, LPO and mPFC were performed according to previously described protocols^10,64^. Mice were treated with buprenorphine (0.1 mg kg^−1^), anesthetized with isoflurane and placed in the stereotactic apparatus (David Kopf Instruments). A small hole was drilled in the skull with a dental drill for each virus injection site according to the stereotactic coordinates. A sterile glass pipette made using a micropipette puller (Sutter Instruments) was mounted on a syringe (Hamilton CS-Chromatographie Service) to infuse viruses at a rate of 100 nl min^−1^; injection volume and speed were controlled with a micro-pump (Harvard Apparatus, Hugo Sachs Elektronik). After the injection, the injection pipette remained in the injection area for about 10 min and then was slowly lifted before the incision was sutured. Optogenetic constructs were purchased from the University of North Carolina (UNC) Vector Core or provided by K. Deisseroth. For manipulation of LH and LPO Vgat cells, Vgat-cre mice were injected bilaterally into the LPO (anterior-posterior (AP) 0 mm, mediolateral (ML) ± 1 mm, dorsal-ventral (DV) −5 mm and −5.25 mm) with 0.3 μl per injection site of AAV8-Ef1a-DIO-ChRmine-mScarlet (provided by K. Deisseroth, titer 5 × 10^12^ vg ml^−1^) or 0.3 μl per injection site of AAV8-Ef1a-DIO-mScarlet (provided by K. Deisseroth, titer 5 × 10^12^ vg ml^−1^). In the LH (AP −1.7 mm, ML ± 1 mm, DV −5 mm and −5.25 mm), 0.3 μl per injection site of AAV2-Ef1a-DIO-ChETA-eYFP (UNC Vector Core, titer 3.5 × 10^12^ vg ml^−1^) or 0.3 μl per injection site of AAV2-EF1a-DIO-eYFP (UNC Vector Core, titer 4.5 × 10^12^ vg ml^−1^) were injected bilaterally. For manipulations of the mPFC–LH or mPFC–LPO projections, C57BL/6 mice were injected bilaterally in the mPFC (AP 1.7 mm, ML ± 0.3 mm, DV −2.4 mm and −2.8 mm) with 0.2 μl per injection site of eNPAC2.0 (provided by K. Deisseroth, titer 1.84 × 10^13^ vg ml^−1^) or AAV5-hSyn-eYFP (UNC Vector Core, titer 3.3 × 10^12^ vg ml^−1^).

### Implantation of optic fibers and electrodes

Optic fiber implants were manufactured from 100-μm diameter multimode optic fiber (numerical aperture 0.22) and zirconia ferrules (Thorlabs). For optogenetic manipulations of signaling between the LH and LPO, mice were implanted with optic fibers in the LH (AP −1.7 mm, ML 1 mm at a 21.8° angle, ML −1 mm, DV −4.7 mm) and in the LPO (AP 0 mm, ML −1 mm at a 21.8° angle, ML 1 mm, DV −4.7 mm). For the optogenetic manipulations of the mPFC–LH projections, mice were implanted bilaterally with optic fibers in the LH (AP −1.7 mm, ML 1 mm at a 21.8° angle, ML −1 mm, DV −4.7 mm). For the optogenetic manipulations of the mPFC–LPO projections, mice were implanted bilaterally with optic fibers in the LPO (AP 0 mm, ML 1 mm at a 21.8° angle, ML −1 mm, DV −4.7 mm). For the extracellular neuronal and LFP recordings, silicon probes (B32, NeuroNexus Technologies) were mounted on custom-made microdrives and implanted as described previously^10,64,65^. For the mPFC and LH simultaneous recordings, the following implantation coordinates were used: mPFC (AP 1.7 mm, ML 0.2 mm, medial shank, DV −2.4 mm) and LH (AP −1.58 mm, ML 0.8 mm, medial shank, DV −4.9 mm). For the LH and LPO recordings, the following implantation coordinates were used: LH (AP −1.58 mm, ML 0.8 mm, medial shank, DV −5 mm) and LPO (AP 0 mm, ML 0.5 mm, medial shank, DV −5 mm) combined with optic fibers implanted at a 21.8° angle in the LH (AP −1.7 mm, ML 1 mm, DV −4.7 mm) and the LPO (AP 0 mm, ML 1 mm, DV −4.7 mm). For the VTA recordings, the following implantation coordinates were used: VTA (AP −3.1 mm, rostral shank, ML 0.4 mm, DV −4.2 mm). For simultaneous LFP recordings from the LH, LPO and VTA, a custom stationary probe (four recording sites × eight shanks, NeuroNexus Technologies; Fig. 7a) was implanted along the line defined by the following coordinates: first shank (AP 0 mm, ML 1 mm, DV 5.4 mm) and last shank (AP −3.8 mm, ML 0.5 mm, DV 4.8 mm).

### Data acquisition

The recording setup was a custom-made enclosure^10^ (length/width/height 50 × 30 × 20 cm) with two interconnected compartments (25 × 30 × 20 cm each). Water presented in a water cup, food provided in a food cup and a new object from Lego or similar toy sets were placed in three corners of the enclosure. Mice were freely moving in the enclosure during the recordings. During the recordings, silicon probes were connected to a preamplifier (NeuraLynx) to eliminate cable movement artifacts. Signals were differentially amplified and band-pass-filtered (1 Hz–8 kHz) and acquired continuously at 32 kHz (Digital Lynx, NeuraLynx). Synchronization with the acquisition of electrophysiological data recording of the animals’ behavior was performed from different angles by four cameras at 25 Hz (Motif, Loopbio). A light-emitting diode was attached to the headset to track the animal’s position at 25 Hz using a top-mounted camera. For pose estimation using DeepLabCut, the behavior of pairs of mice in the enclosure was recorded at 15 Hz. For behavioral motion segmentation (MoSeq^21^), female mice were recorded for 20 min while they were freely exploring an arena (length/width/height 45 × 25 × 40 cm) with a female conspecific and either a new object (piece of Lego) or (high-fat) food behind a mesh (length/width/height 8 × 8 × 7 cm) on the left and right sides, respectively. Behavior in these experiments was captured at 30 Hz with a depth camera (Kinect for Windows v.2, Microsoft) positioned 65 cm above the floor of the arena.

### Optogenetic stimulation

All mice used in the behavioral assays were allowed to recover after the fiber implantation for at least 1 week. Mice were randomly assigned to the experimental conditions. For optogenetic manipulation, 473 nm and 589 nm diode-pumped solid-state lasers (Laserglow Technologies) were used. For the stimulation of projections of LPO cells expressing ChRmine, a light delivery from a 589-nm laser was controlled by a shutter (Doric Lenses). Stimulation protocols were implemented using a stimulus generator (Multi Channel Systems). One side of the patch cord was connected to the implanted optical fiber with a zirconia sleeve (components from Thorlabs) and the other side was connected to the laser with an FC/PC adapter. The optogenetic experiments were performed in the test enclosure described above, once for each type of the stimulation and 14 ± 7 times for different types of optogenetic experiments lasting approximately 20 min each. During the electrophysiological recordings (38 ± 5 sessions per mouse), time stamps of laser pulses were acquired synchronously with neuronal signals and video frames. The behavior of mice was recorded from different angles by four cameras at 25 Hz (Motif, Loopbio).

### Optogenetic manipulations of the LH–LPO circuit

For a closed-loop optogenetic manipulation of the LH–LPO circuit in mice expressing ChRmine-mScarlet in LPO Vgat cells and ChETA-eYFP in LH Vgat cells, beta out-of-phase, nonrhythmic or beta in-phase stimulation was applied unilaterally (Extended Data Fig. 6a–e). A separate control group of mice expressing mScarlet in LPO Vgat cells and eYFP in LH Vgat cells, without optogenetic actuators, also received closed-loop stimulation with the beta out-of-phase protocol. Beta out-of-phase stimulation consisted of 5-ms 589-nm light pulses in the LH and 5-ms 473-nm light pulses in the LPO at 20 Hz with a 25-ms offset between brain regions. During nonrhythmic stimulation, the amount of light irradiation was matched to a 10% duty cycle of the beta out-of-phase and in-phase protocols: 20 pulses, 5 ms each, were randomly assigned times outside the beta band (mean interpulse interval 5 ms) during 200-ms epochs of each 1-s window. These 200-ms epochs of the 589-nm stimulation in the LH and 473 nm in the LPO did not overlap. The light power output was 1–4 mW during the light pulses measured at the tip of each of the two patch cords using an optical power meter (Thorlabs). Beta out-of-phase, nonrhythmic or beta in-phase stimulation was started when an animal spontaneously initiated F, S or E, in separate experiments, and lasted for 5 or 10 s for each manipulated behavioral episode during four corresponding 5-min parts of a 20-min session (Extended Data Fig. 6i). The stimulation was repeated each time when an animal engaged in the investigated behavior. The time elapsed from stimulation onset to the end of the behavioral episode was defined as the latency to behavioral transition.

Noncontingent on the animals’ behavior, either beta out-of-phase or unidirectional LPO–LH stimulation (Extended Data Fig. 6d) was applied for 20 min in repeated blocks of 10-s stimulation alternating with 20-s breaks.

### Optogenetic manipulations of mPFC–LH and mPFC–LPO projections

In mice expressing eNPAC2.0-eYFP or eYFP in the mPFC, stimulation was performed in the LH or in the LPO. Each experimental session lasted for 20 min. Pulses of 473-nm light for 5 ms at 20 Hz, a light power output of 10–15 mW from the tip of the patch cord or 589-nm light for 10 s continuously and a light power output of 20 mW from the tip of the patch cord were applied in the optogenetic excitation or inhibition experiments, respectively. The closed-loop optogenetic stimulation was triggered by the spontaneous initiation of behaviors and lasted for 10 s for each manipulated behavioral episode. For the social behavior tests, stimulation at theta frequency (9 Hz: 11-ms light-on, 100-ms light-off phases for 10 s in each stimulation episode) and nonrhythmic stimulation with light intensity matched to the beta frequency protocol (as described for the LH–LPO circuit stimulation) were performed.

### Brain dissection and imaging

After completion of the experiments, mice were deeply anesthetized and electrolytic lesions at selected recording sites were performed to visualize the locations of the recording electrodes. Mice were perfused with 4% paraformaldehyde in PBS. Brains were fixed overnight in paraformaldehyde, placed for cryoprotection in 30% sucrose at 4 °C for 24 h and then coronally or sagittally sectioned into 40-μm slices on a cryostat (CM1900, Leica Biosytems). To visualize the electrolytic lesions and silicon probe tracks, brain sections were imaged using a widefield Axio Imager M2 microscope (ZEISS). To visualize the projections and control viral expression, sections were imaged using a confocal microscope (Leica SP8, Leica Biosytems).

### Behavioral analysis

#### Behavioral scoring

Ethograms were obtained using a frame-by-frame scoring of behaviors using Adobe Premiere Pro (v.2020) (Adobe) in multiangle synchronized video recordings^66^. Frames when a resident mouse (implanted with electrodes or optic fibers) was consuming food pellets were scored as feeding. Social contact was defined as sniffing or following an intruder mouse. During the stimulation of the LH–LPO circuit, the latter behavior evolved into a prolonged chasing, defined as uninterrupted pursuing of an intruder for longer than 2 s. New object exploration was defined as sniffing, gnawing, touching or climbing a new object.

#### DeepLabCut

Markerless pose estimation was performed with the DeepLabCut toolbox (v.2.2.0.2)^22^. First, *k*-means clustering and manual selection were performed to select frames from each video across behaviors. Six key points (snout, left ear, right ear, left side (middle-left part of body), right side (middle-right part of body) and tail base) of each animal were localized on each frame. In total, 610 labeled frames were selected across eight video recordings and used to train a multiscale deep learning model DLCR-Net_ms5. Randomly assigned 95% of the data were used for training and the rest for testing. The network was trained for 120,000 iterations until cross-entropy loss plateaued. The estimated coordinates of key points in each frame were used to define behaviors^67^: animal in the food zone (the distance between the food zone center and the snout or an ear was less than the radius of food zone, 3.6 cm); feeding (snout and both ears in the food zone for at least 1.3 s); mouse in the water zone (the distance between the water zone center and the snout or an ear was less than the radius of the water zone, 3 cm); drinking (snout in the water zone for at least 1.5 s); social contact (snout or an ear inside or on the edge of the polygon area defined by the six key points of an intruder mouse); new object exploration (the distance between the new object zone and the snout or an ear was less than 1.3 cm); rearing (the snout was at least 2.2 cm over the enclosure wall or the snout was at least 0.7 cm over the middle separator wall and the length of a vector between the snout and the tail base was less than 10.3 cm); and immobility (the coordinates of each of the six key points changed less than 0.5 cm s^−1^). Frames with pose patterns not meeting any of these criteria were classified as behaviorally undefined.

#### Behavioral motion segmentation

Using custom Python scripts (adapted from MoSeq (v.1)^21^ by R. Ung from the G. Stuber’s laboratory), depth images and frame time stamps were converted into a binary format for further analysis. Region-of-interest polygons delimiting the boundaries of the arena and two-dimensional images to inspect the behavioral syllables after analyses were acquired simultaneously.

MoSeq was performed in a Debian GNU/Linux 8 virtual environment running on a Linux (Ubuntu 16.94.3 LTS) compute cluster. Behavior was classified using MoSeq v.1 (ref. ^21^). Briefly, depth mouse images were cropped along the arena boundaries, extracted from the arena background, parallax-corrected and orientated along the spine axis. Time series data were subjected to wavelet transformation and dimensionally compressed using principal component analysis. To classify the behavioral syllables, an autoregressive hidden Markov model was applied to the first ten principal components. A template-matching procedure ensured that only repeated principal component trajectories (that is, meaningful ones) were selected as syllables. One of the model parameters, the self-transition bias kappa, was set to match the median syllable duration with the median approximate change point of each dataset identified using a filtered derivative algorithm (*κ* = 5). To qualitatively verify behavioral syllables, we manually assessed the visualizations of each syllable using the two-dimensional recordings.

Analyses of syllable usage were performed in Python v.2.7. A narrow zone of 10 × 5 cm before the mesh with food, conspecific or new object was defined as a contact zone. The 4-s periods just before entering the zone (with the center of the head), with a minimum zone visit duration of 333 ms and a minimum zone visit interval of 2 s, was divided into eight 0.5-s bins (hence, for example, ‘−2 s’ corresponds to the period from 2 s to 1.5 s before social contact). Frames corresponding to the previous zone visit were excluded from the transition periods. For the calculation of syllable usage during contact, all frames inside the contact zone were included. The probability of syllable usage during randomly selected 2-s epochs and repeated 1,000 times, equal or greater than the usage during the transition epochs, was computed and normalized across bins.

### Electrophysiological data analysis

#### Spike sorting and unit characterization

Electrophysiological signals were preprocessed using NDManager (http://neurosuite.sourceforge.net/)^68^ and analyzed using custom-written MATLAB v.2014b algorithms (MathWorks) as described previously^64^. Action potentials (spikes) were detected in a high-pass filtered signal and spike waveforms were represented by the first three principal components and by the amplitudes of the action potentials. Spike sorting was performed automatically^69^ (https://github.com/klusta-team/klustakwik) followed by manual cluster adjustment based on auto-correlations and cross-correlations of spikes trains, the Mahalanobis distance between pairs of clusters and the visual comparison of waveform profiles across channels^68^ (Extended Data Fig. 1b). Isolation distance^69^ was computed for the sorted units: LH = 62.5 ± 0.9, *n* = 2,417 cells; LPO = 63.8 ± 2.0, *n* = 415 cells; mPFC = 66.9 ± 1.1, *n* = 2,374 cells; VTA = 82.0 ± 3.2, *n* = 308 putative dopamine cells.

For individual behaviors, we computed the firing rate of cells. A surrogate distribution of 1,000 firing rate values was obtained for each cell by 2–4-min offsets of the behavior time stamps. For each behavior, the match score was calculated as the percentile of the firing rate during the behavior in the surrogate distribution. Multimodal LH cells were defined as units with a firing rate preference in the upper quartile for each of the three behaviors.

#### LFP analysis

The LFP was obtained by downsampling the wide band signal to 1,250 Hz using NDManager^68^. High-resolution time frequency analysis was performed using a continuous Morlet wavelet transform. The multitaper method (NW = 3, window length of 1,024) was used to compute power spectral density and coherence according to the ethogram times. Beta oscillations were detected in the 15–30 Hz band-pass-filtered, rectified and smoothed signal. Events with amplitudes exceeding 2 s.d. above the noise mean for at least 80 ms were detected. The beginning and the end of the oscillatory epochs were designated at times when the amplitude fell below 1 s.d.

#### Discharge phases

Spikes fired during the detected oscillation episodes were assigned beta oscillation phases, computed using the Hilbert transform of the 15–30 Hz filtered signal. Histograms of spike counts in 20 phase bins were convolved with a Gaussian kernel (size = 0.65 s.d.) and normalized by the total number of spikes in the histogram^65^. This approach was also used to compute the discharge phases during the gamma oscillations (30–60 Hz, minimum duration of 25 ms (ref. ^10^).

To examine the timing of neuronal discharge in the LH and LPO during beta out-of-phase stimulation, we used a linear approximation of the 20-Hz sinewave as a reference for the spike phase assignment. The times of blue light pulses stimulating projections of LH cells in the LPO and of red light pulses stimulating projections of LPO cells in the LH defined the period of the stimulation rhythm for the assignment of phases to the spikes of LH and LPO cells, respectively. As we optogenetically stimulated the inhibitory inputs from the LH to the LPO and from the LPO to the LH, we evaluated the proportion of units inhibited by the optogenetic stimulation. For this purpose, we summed the normalized binned firing probability within the first 7 ms after pulse termination and the normalized binned firing probability within the following 7 ms. We calculated the ratio of these sums and detected any outlier units defined as more than three scaled median absolute deviations away from the median. Units falling below the 30th percentile in the ratio distribution were defined as inhibited units, the population firing probability of which was summarized in stimulation phase histograms. The onset of the first stimulation pulse was assigned as phase *π* and the onset of every second pulse was assigned as phase −*π*. Every mid-interpulse interval was assigned as phase 0 radian. Other phases were linearly interpolated at 20 kHz (the sampling rate of spike trains). Each spike was assigned a corresponding beta phase in the stimulation cycle. The obtained phases were offset by *π* for the LH spike strains (that is, stimulation of the LH cell projections at 0° and 360°) and by 3 *π* for the LPO spike trains (that is, stimulation of the LPO cell projections at 180° and 520°) according to the out-of-phase timing of the pulses in this stimulation protocol. The spike phase distribution of each unit was binned into 20 bins per beta cycle.

### Machine learning modeling

#### Phase signatures

Firing probability versus beta oscillation phase histograms were computed for individual LH and VTA cells using the spikes fired during 2 s before transition to F, S and E separately for each behavior for control or behavior epochs of the same duration. Control epochs excluded transitions to the aforementioned behaviors. In a separate analysis, transition and control epochs were additionally selected for the same behavioral state, locomotion and posture change.

Cells with histograms containing at least 20 (168 ± 15) spikes for the LH cells and at least 10 (29 ± 3) spikes for the presumed dopamine cells (VTA cells with a spike width greater than 0.3 ms (ref. ^29^) and firing rate lower than 10 Hz (ref. ^47^) were used for the subsequent decoding of behavioral transitions. For each of the 20 phase bins, the population distribution of firing probabilities was estimated; cells with the firing probability in the upper quartile of the distribution, that is, ‘highly active cells’ at a given phase, were selected. A phase signature was defined through an asymmetry of individual behavior match scores’ distribution in a population of highly active cells in each phase bin as:

$$\varphi :=\frac{c}{N-c}$$

where $c=|\{m\in D:m > 0.5\}|$ and *D* is a set of match scores *m* of *N* cells.

Phase signatures were computed based on match scores for F, S and E during transitions to these three behaviors resulting in three behavior-specific phase signatures for each of the three types of transitions or, if specified, for a combination of different types of transitions. To account for the variability of the phase signatures in each phase bin, the distribution of match scores in the set of highly active cells at a given phase was bootstrapped with replacement 1,000 times to derive the datasets for the modeling^70^. To generate the control sets, the time stamps of the transitions were randomly offset, excluding overlaps with the transition epochs from native ethograms. The first 1,000 offset trials with the number of spikes that were sufficient for the estimation of firing probability and the phase histograms were selected.

#### Support vector classifications

SVM models were implemented using the Python package Scikit-learn (v.1.2.2)^71^. Phase signatures from either individual or multiple phase bins were the inputs to one SVM. The classes and input datasets for the SVMs are described below (see also the design description of the SVM model in Supplementary Information).

Models 1 and 2 in Fig. 2a–c aimed to classify transition (2-s epochs) versus control epochs (2 s, excluding transitions to the three behaviors) within individual phase bins (eight bins) in the peak neighborhood (peak ± 72°). To do so, an SVM was trained and tested with tenfold cross-validation. Model 1 was trained and tested on phase signatures related to individual behaviors (F, S and E), while model 2 used the phase signatures of all three behaviors in one SVM.

Model 3 in Fig. 2d assessed the phase signatures (related to F, S and E) in transition versus control epochs across phases near the peaks of beta oscillations. To generate the phase-shuffled datasets, the phase of each spike was jittered by a random offset from a uniform distribution. Then all spikes of all cells were additionally offset by the same random phase between 2.5 radian (the width of the peak neighborhood) and *π* using different random offsets for control and transition epochs. Separate SVMs were computed on phase signatures (related to F, S and E) from the original and phase-shuffled datasets to classify transition versus control epochs. Training was done on the data from the phase bin with the highest (in the entire oscillation cycle) difference of phase signature amplitude between transition and control. Training was performed this way in the original and in the phase-shuffled data. Subsequently, the SVM trained on the original dataset was tested on individual phase bins in the peak neighborhood in the original dataset, excluding the bin used for training. Testing in that bin was performed using a separate SVM, trained on the phase bin with the second highest (in the entire oscillation cycle) amplitude phase signature. Testing of the SVM trained on phase-shuffled data was performed on the phase-shuffled data from the bin, in which the original dataset could be decoded with the highest accuracy. The resulting decoding accuracy in the phase-shuffled data was close to the chance level, which was typical for phase-shuffled data also in other phase bins.

Model 4 in the statistical information in Supplementary Information for Fig. 3c and Extended Data Fig. 5b classified three upcoming behaviors (F, S and E) using in one SVM phase signatures from all eight phase bins in the peak neighborhood during transitions. The SVMs were trained and tested on the phase signatures of individual behaviors (F, S and E) with tenfold cross-validation.

Model 5 in the statistical information in Supplementary Information for Fig. 3c and Extended Data Fig. 5b was similar to model 4 except that, instead of upcoming behaviors, it classified three current behaviors (F, S and E) using the phase signatures during random 2-s epochs of behaviors.

Models 6 and 7 in Fig. 3d were similar to models 1 and 2, respectively, but they classified three upcoming behaviors (F, S and E) using the phase signatures in individual phase bins in the peak neighborhood during transitions.

Models 8 and 9 in Fig. 3e were similar to models 1 and 2, respectively, but they classified three current behaviors (F, S and E) using the phase signatures in individual phase bins in the peak neighborhood. These SVMs were trained and tested on phase signatures during random 2-s epochs of behaviors (F, S and E).

Model 10 in Fig. 7i classified transition (2-s epochs) versus control epochs (2 s, excluding transitions to the above behaviors) using in one SVM the phase signatures from all phase bins in the entire cycle to account for the phase offset of dopamine cell discharge in relation to the LH (LH in Fig. 4a; VTA^dopamine^ in Extended Data Fig. 10d).

Models 11 and 12 in Extended Data Fig. 4d,e were similar to model 3 except that they classified pooled transitions (2-s epochs preceding any of the three behaviors, that is, F, S and E) versus control epochs (2 s, excluding transitions to the three behaviors), either using the data from all mice pooled (model 11) or separately from individual mice (model 12).

In the LH recordings, as described above, two-class and three-class (one-versus-rest multiclass classification^72^) models were computed using a nonlinear radial basis or linear kernel (depending on the dimensionality of the feature space). Linear SVMs were used to classify the population activity of putative dopamine cells. To minimize overfitting, training and testing were done on different data subsets. Except for models 3, 11 and 12, which were designed to be trained and tested on different phase bins, a stratified tenfold cross-validation procedure was used: each training set was randomly divided into ten subsamples with the same proportion of samples from each class as in the complete set. One subsample was then retained for testing the model, while the other nine subsamples were used for training, with this procedure repeated using all ten subsamples so that each subsample was used only once to evaluate the performance of the model. Decoding accuracies were initially averaged within tenfold cross-validation trials and the resulting accuracies were averaged across 1,000 repeated cross-validations.

The significance of classifications was assessed using permutation tests. We randomly permuted the labels and then used the same decoding approach as for decoding the original labels, except for using the stratified tenfold cross-validation once (instead of 1,000 times) for each model. The permutation of labels was repeated 1,000 times to assess the chance performance of a classifier computed as the average of accuracies across permuted sets. The performance of a classifier was considered significant when it fell in the 5% upper tail of its chance performance distribution.

### Statistical analyses

Statistical analyses were performed using MATLAB v.2014b (MathWorks), Python v.3 (https://www.python.org/) or Prism 9 (GraphPad Software). The level of significance and the number of neurons and mice are indicated in the figure legends. A likelihood ratio test^73^ was used to compare bivariate circular distributions (see the statistical information related to Fig. 5d,e). All statistical tests were two-tailed unless indicated otherwise; permutation and randomization tests were right-tailed. Two-group comparisons were performed using a *t*-test, Mann–Whitney *U*-test or Wilcoxon matched-pairs test depending on the normality of a distribution. Multiple group comparisons were performed using an ANOVA or multiple two-group tests with *α* correction, adjusting for multiple comparisons. The Grubbs’ test was used to exclude outlier points from behavioral datasets. A median absolute deviation outlier test was used to exclude outlier points from the analysis of optogenetic entrainment. No further data points or animals were excluded. Sample size was determined according to the accepted practice for the applied assays. No statistical methods were used to predetermine sample sizes; sample sizes are similar to those reported in previous publications^38,49,54,66^. Data analysis was performed blindly using automatic selection of data from a database. The full description of the statistical analyses corresponding to each dataset is provided in the statistical information in Supplementary Information. Unless specified otherwise, descriptive statistics are reported as the mean ± s.e.m.

### Reporting summary

Further information on research design is available in the Nature Portfolio Reporting Summary linked to this article.

## Online content

Any methods, additional references, Nature Portfolio reporting summaries, source data, extended data, supplementary information, acknowledgements, peer review information; details of author contributions and competing interests; and statements of data and code availability are available at 10.1038/s41593-024-01598-3.

### Supplementary information

Supplementary Information Supplementary Methods and statistical information.

Reporting Summary

**Supplementary Videos 1 and 2**

Supplementary Videos 1 and 2. Elimination of behavioral transitions by a phase-specific intrahypothalamic inhibition during feeding. Beta out-of-phase optogenetic stimulation of the LH–LPO circuit was applied in two representative mice, expressing ChRmine-mScarlet in the LPO Vgat cells and ChETA-eYFP in the LH Vgat cells. The stimulation was applied upon a spontaneous onset of feeding and lasted 10 s (Supplementary Video 1) or until the end of the feeding episode (Supplementary Video 2). Multiangle recording of the same enclosure. The stimulation epoch is indicated with ‘Laser ON’. The LED lights correspond to the alternating activation of blue and red lasers.

**Supplementary Videos 3 and 4**

Supplementary Videos 3 and 4. Elimination of behavioral transitions by a phase-specific intrahypothalamic inhibition during social contact. Beta out-of-phase optogenetic stimulation of the LH–LPO circuit was applied for 10 s in two representative mice, expressing ChRmine-mScarlet in the LPO Vgat cells and ChETA-eYFP in the LH Vgat cells upon a spontaneous onset of social contact with an intruder mouse. Multiangle recording of the same enclosure. The stimulation epoch is indicated with ‘Laser ON’. The LED lights correspond to the alternating activation of blue and red lasers.

Supplementary Video 5 Supplementary Video 5. A behavioral transition evoked by the optogenetic activation of mPFC–LH projections during feeding. Beta-rhythmic optogenetic stimulation of the mPFC–LH projections was applied in the LH of a representative mouse, expressing eNPAC2.0 in mPFCs, upon spontaneous onset of feeding. Multiangle recording of the same enclosure. The stimulation epoch is indicated with ‘Laser ON’.

Supplementary Video 6 Supplementary Video 6. A behavioral transition evoked by the optogenetic activation of mPFC–LH projections during social contact. Beta-rhythmic optogenetic stimulation of the mPFC–LH projections was applied in the LH of a representative mouse, expressing eNPAC2.0 in mPFCs upon a spontaneous onset of social contact with an intruder mouse. Multiangle recording of the same enclosure. The stimulation epoch is indicated with ‘Laser ON’.

### Source data

Source Data Fig. 1 Statistical source data.

Source Data Fig. 2 Statistical source data.

Source Data Fig. 3 Statistical source data.

Source Data Fig. 4 Statistical source data.

Source Data Fig. 5 Statistical source data.

Source Data Fig. 6 Statistical source data.

Source Data Fig. 7 Statistical source data.

Source Data Extended Data Fig. 1 Statistical source data.

Source Data Extended Data Fig. 2 Statistical source data.

Source Data Extended Data Fig. 3 Statistical source data.

Source Data Extended Data Fig. 4 Statistical source data.

Source Data Extended Data Fig. 5 Statistical source data.

Source Data Extended Data Fig. 6 Statistical source data.

Source Data Extended Data Fig. 7 Statistical source data.

Source Data Extended Data Fig. 8 Statistical source data.

Source Data Extended Data Fig. 9 Statistical source data.

Source Data Extended Data Fig. 10 Statistical source data.

## Data Availability

Source data for Figs. 1–7 and Extended Data Figs. 1–10 are provided in the paper. Spike trains recorded in the LH, mPFC, VTA and the time stamps of beta oscillations have been made available via Figshare (10.6084/m9.figshare.22317091). Further datasets generated during the current study are available from corresponding authors upon reasonable request. Source data are provided with this paper.

## Code availability

All code used in the current study is available via GitHub (https://github.com/NeuroAnalyz/SpikeTrains-LFP) or from the corresponding authors upon reasonable request.
