# Supplementary material for: The dynamic state of a prefrontal–hypothalamic–midbrain circuit commands behavioral transitions
Source: Nat Neurosci. 2024 Mar 18;27(5):952–63. doi: 10.1038/s41593-024-01598-3 (PMC11089001; doi:10.1038/s41593-024-01598-3)
Supplement: Supplementary file 1 — Supplementary Methods and statistical information. [file 41593_2024_1598_MOESM1_ESM.pdf]

nature neuroscience

Article https://doi.org/10.1038/s41593-024-01598-3

The dynamic state of a prefrontal–hypothalamic–midbrain circuit commands behavioral transitions

In the format provided by the authors and unedited

Supplementary Methods

SVM's models' design

Fig. 2a, b, c (violet, purple and peach). Class labels: two sec long transition epochs preceding individual behaviours (F, S, E) vs. control epochs, non-overlapping with the transition epochs. **Input data**: phase signatures of individual behaviours (F, S, E). **Beta phases**: training and testing on each individual phase bin in the peak neighborhood.

Fig. 2a, b, c (yellow, "All"). Class labels: two sec long transition epochs preceding individual behaviours (F, S, E) vs. control epochs, non-overlapping with the transition epochs. **Input data**: phase signatures of multiple behaviours (F, S, E). **Beta phases**: training and testing on each individual phase bin in the peak neighborhood.

Fig. 2d. Class labels: two sec long transition epochs preceding individual behaviours (F, S, E) vs. control epochs, non-overlapping with the transition epochs. **Input data**: phase signatures of individual behaviours (F, S, E). **Beta phases**: training on phase bin with the highest (or the second highest, for decoding the former bin) difference of phase signatures amplitude during transition epochs compared to control epochs; Testing for original data on individual phase bins in the peak neighborhood and testing for phase-shuffled data on the bin corresponding to the highest accuracy in the original data.

Fig. 3c, Extended data Fig. 5b (left). Class labels: two sec long epochs of transition preceding to F vs. preceding to S vs. preceding to E. **Input data**: phase signatures of individual behaviours (F, S, E). **Beta phases**: training and testing on all phase bins in the peak neighborhood, combined.

Fig. 3c, Extended data Fig. 5b (right). Class labels: two sec long epochs of three current behaviours F vs S vs E. **Input data**: phase signatures of individual behaviours (F, S, E). **Beta phases**: training and testing on all phase bins in the peak neighborhood, combined.

Fig. 3d (grey, top). Class labels: two sec long epochs of transition preceding to F vs. preceding to S vs. preceding to E. **Input data**: phase signatures of individual

behaviours (F, S, E). **Beta phases**: training and testing on each individual phase bin in the peak neighborhood.

Fig. 3d (green, right). Class labels: two sec long epochs of transition preceding to F vs. preceding to S vs. preceding to E. **Input data**: phase signatures of multiple behaviours (F, S, E). **Beta phases**: training and testing on each individual phase bin in the peak neighborhood.

Fig. 3e (white, top). Class labels: two sec long epochs of three current behaviours F vs. S vs. E. **Input data**: phase signatures of individual behaviours (F, S, E). **Beta phases**: training and testing on each individual phase bin in the peak neighborhood.

Fig. 3e (brown, right). Class labels: two sec long epochs of three current behaviours F vs. S vs. E. **Input data**: phase signatures of multiple behaviours (F, S, E). **Beta phases**: training and testing on each individual phase bin in the peak neighborhood.

Fig. 7i. Class labels: two sec long transition epochs preceding multiple behaviours (F, S, E) combined vs. control epochs, non-overlapping with the transition epochs. **Input data**: phase signatures of individual behaviours (F, S, E). **Beta phases**: training and testing on all phase bins in entire cycle, combined.

Extended data Fig. 4d, e. Class labels: two sec long transition epochs preceding multiple behaviours (F, S, E) combined vs. control epochs, non-overlapping with the transition epochs. **Input data**: Phase signatures of individual behaviours (F, S, E) based on cells from different mice combined (**d**) or from individual mice (**e**). **Beta phases**: training on individual phase bin with the highest (or the second highest, for decoding the former bin) difference of phase signatures amplitude during transition compared to control epochs;
testing for original data on individual phase bins in the peak neighborhood and testing for phase-shuffled data on the bin corresponding to the highest accuracy in the original data.

Supplementary Statistical Information

In Fig. 1f, **3a**, **4d**, **7g**, **Extended Data Fig. 3b**, **3c**, **3d and 7a** phase signatures were convolved with a Gaussian kernel of size 3, SD 2 and averaged across 1000 bootstrap (transitions, behaviour) and shuffling (control) sessions.

For Fig. 1a. Instantaneous firing rate was estimated by the convolution of spike trains with a gaussian kernel of the size of 500 ms.

For Fig. 1b. Firing rate estimation of the LH population in relation to feeding episodes scaled for each cell from the maximum to the minimum. Firing rate curves were sorted in the ascending order of average values. The duration of feeding episodes did not differ between consecutive sessions, one-way ANOVA, feeding: $F_{2,8} = 0.1$, $p = 0.9$.

For Fig. 1c. Pearson partial correlations of normalized match scores for different behaviours within individual 105 LH cells: feeding and social contact, r = 0.01, p = 0.9; feeding and novel object expl., r = -0.13, p = 0.2; social contact and novel object expl., r = 0.15, p = 0.1; feeding and locomotion, r = 0.09, p = 0.4; feeding and immobility, r = -0.20, p = 0.04; novel object expl. and locomotion, r = 0.10, p = 0.3; novel object expl. and immobility, r = -0.13, p = 0.2, social contact and locomotion, r = 0.63, p < 0.0001; social contact and immobility, r = -013, p = 0.2 (correlation of phase signatures related to social contact and locomotion for combined transitions, r = -0.25, p = 0.5).

For Fig. 2a-c. Accuracies of decoding transitions to different behaviours using phase signatures to individual behaviours and their combination, n = 8 phase bins. Transition to Feeding, paired t-test, adjusted α = 0.0167: φ_F vs. All, $t_7 = 4.2$, p = 0.004, φ_S vs. All, $t_7 = 3.8$, p = 0.006, φ_E vs. All, $t_7 = 2.8$, p = 0.02. Transition to Social contact, paired t-test, adjusted α = 0.0167: φ_F vs. All, $t_7 = 5.0$, p = 0.002, φ_S vs. All, $t_7 = 8.4$, p < 0.0001, φ_E vs. All, $t_7 = 5.3$, p = 0.001. Transition to Obj.expl., paired t-test, adjusted α = 0.0167: φ_F vs. All, $t_7 = 3.6$, p = 0.008, φ_S vs. All, $t_7 = 5.4$, p = 0.00096, φ_E vs. All, $t_7 = 3.2$, p = 0.0149.

For Fig. 2d. Randomization tests, probability of observing the decoding accuracies (averaged across F, S, E phase signatures) in the 1000 phase-shuffled datasets equal or greater than the decoding accuracy in the original dataset. Transition to feeding: $p = 0.0009$, to social contact: $p = 0.0019$, to novel object exploration: $p = 0.0009$.

For Fig. 2e. Impact of behavioural states on the decoding of transitions. SVM decoding using phase signatures of multiple behaviours computed during transitions to feeding, social contact and novel object exploration (types of transitions combined, bootstrap) during locomotion and postural change (prevalent behavioural state during transitions) vs. the phase signatures during 1000 shuffled sets of control locomotion and postural change epochs excluding transitions, accuracy of 79 ± 2 %, mean ± SEM across eight phase bins in the peak neighborhood.

For Fig. 3b. Peak/trough preference of averaged across the three behaviours phase signatures computed prior transitions, during transitions and after behaviour onset. Unpaired t-test, adjusted $\alpha = 0.025$, Prior to transition vs. Transition: $t_{1998} = 35.5$, $p < 0.0001$, Behaviour vs. transition: $t_{1998} = 98.5$, $p < 0.0001$.

For Fig. 3c. SVM accuracies of decoding upcoming behaviour using individual behaviour phase signatures in eight phase bins in the peak neighborhood, computed during transitions, fed to the same SVM model: (φ_F): 93% and (φ_S): 81%. Decoding current behaviour using individual behaviour phase signatures in the peak neighborhood fed to the same SVM model: (φ_F): 41% and (φ_S): 39%. Permutation test for significance of the classifiers for decoding upcoming behaviour: $p < 0.0001$ for φ_F and φ_S, for decoding current behaviour: $p < 0.0001$ for φ_F and $p = 0.002$ for φ_S (for details see Methods, Machine learning modelling).

For Fig. 3f, **g**, Decoding of upcoming vs. current behaviours using phase signatures of individual behaviours, paired t-test, adjusted $\alpha = 0.0167$: (φ_F) $t_7 = 4.4$, $p = 0.003$, (φ_S) $t_7 = 5.8$, $p = 0.0007$, (φ_E) $t_7 = 5.8$, $p = 0.0007$. Decoding upcoming vs. current behaviours using phase signature of multiple behaviours, paired t-test, $t_7 = 10.30$, $p < 0.0001$.
Comparison of accuracies of decoding upcoming behaviour using phase signatures of individual vs. multiple (All) behaviours, n = 8 phase bins. Paired t-test, adjusted $\alpha = 0.0167$: φ_F vs. All, $t_7 = 7.3$, $p = 0.00016$, φ_S vs. All, $t_7 = 5.3$, $p = 0.001$, φ_E vs. All, $t_7 = 4.6$, $p = 0.002$.
Comparison of accuracies of decoding current behaviour using phase signatures of individual vs. multiple (All) behaviours, n = 8 phase bins. Paired t-test, adjusted $\alpha = 0.0167$: φ_F vs. All, $t_7 = 1.9$, $p = 0.09303$, φ_S vs. All, $t_7 = 2.5$, $p = 0.038$, φ_E vs. All, $t_7 = 2.6$, $p = 0.036$.

Permutation test for significance of the classifiers, separately for each model/phase bin, decoding upcoming behaviours: $p < 0.0001$ for all the bins, decoding current behaviours: $p < 0.05$ for all the bins (for details see Methods**,** Machine learning modelling).

For Fig. 4c. Average firing probability of LH cells vs. their firing probability during blue light stimulation of LH projection in the LPO, one-sample t-test, $t_{51} = 0.53$, $p = 0.6$. Average firing probability of LH cells vs. their firing probability during red light stimulation of LPO projections to the LH, one-sample t-test, $t_{51} = 7.76$, $p < 0.0001$. Firing probability of LH cells during the application of blue (0.051 ± 0.003, mean ± SEM) vs. red (0.037 ± 0.002) light, paired t-test, $t_{51}=4.87$, $p < 0.0001$.

For Fig. 4f. Significance of the beta oscillation peak preference of averaged across the three behaviours phase signatures, Ho: peak/trough preference of the average phase signature is equal or less than one (1 - a uniform phase signature), bootstrap test, $p = 0.84$.

For Fig. 4h. Latency to transition during 10 s (epochs 1 and 3) or 5 s (epochs 2 and 4) optogenetic stimulation during feeding. N = 6 (YFP), 6 (Con.), 6 (Stim.) mice. Unpaired t-test, adjusted $\alpha = 0.0063$. Epoch 1, YFP vs. Stim.: $t_{10} = 7.6$, $p < 0.0001$. Con. vs. Stim.: $t_{10} = 6.2$, $p < 0.0001$. Epoch 2, YFP vs. Stim.: $t_{10} = 10.3$, $p < 0.0001$. Con. vs. Stim.: $t_{10} = 8.7$, $p < 0.0001$. Epoch 3, YFP vs. Stim.: $t_9 = 20.9$, $p < 0.0001$. Con. vs. Stim.: $t_9 = 7.2$, $p < 0.0001$. Epoch 4, YFP vs. Stim.: $t_{10} = 4.7$, $p = 0.0008$. Con. vs. Stim.: $t_{10} = 5.1$, $p = 0.0005$. For Stim., paired t-test, epoch 1 vs. epoch 2: $t_5 = 5.0$, $p = 0.004$. Epoch 1 vs. Epoch 3: $t_4 = 1.9$, $p = 0.13$. Epoch 1 vs. Epoch 4: $t_5 = 4.2$, $p = 0.0086$. Epoch 2 vs. Epoch 3: $t_4 = 15.4$, $p = 0.0001$. Epoch 2 vs. Epoch 4: $t_5 = 0.6$, $p = 0.55$. Epoch 3 vs. Epoch 4: $t_4 = 8.7$, $p = 0.001$.

For Fig. 4i. The difference of latency to transition between 10- and 5-second-long stimulation epochs during feeding. N = 6 (YFP), 6 (Con), 6 (Stim) mice. Unpaired t-test, adjusted $\alpha = 0.025$. YFP vs. Stim: $t_{10} = 7.1$, $p < 0.0001$. Con. vs. Stim.: $t_{10} = 4.8$, $p = 0.0007$.

For Fig. 4j. Latency to transition during 10 s (epochs 1 and 3) or 5 s (epochs 2 and 4) optogenetic stimulation during social contact. N = 6 (YFP), 6 (Con.), 6 (Stim.) mice. Unpaired t-test, adjusted $\alpha = 0.0063$. Epoch1, YFP vs. Stim.: $t_{10} = 9.2$, $p < 0.0001$. Con. vs. Stim.: $t_{10} = 8.9$, $p < 0.0001$. Epoch 2, YFP vs. Stim.: $t_9 = 5.7$, $p = 0.0003$. Con. vs. Stim.: $t_{10} = 6.0$, $p = 0.0001$. Epoch 3, YFP vs. Stim.: $t_{10} = 5.8$, $p = 0.0002$. Con. vs. Stim.: $t_{10} = 6.3$, $p < 0.0001$. Epoch 4, YFP vs. Stim.: $t_9 = 8.2$, $p < 0.0001$. Con. vs. Stim.: $t_{10} = 9.1$, $p < 0.0001$. For Stim., paired t-test, epoch 1 vs. epoch 2: $t_5 = 3.3$, $p = 0.02$. Epoch 1 vs. epoch 3: $t_5 = 0.9$, $p = 0.4$.

Epoch 1 vs. epoch 4: $t_5 = 4.2$, $p = 0.0082$. Epoch 2 vs. epoch 3: $t_5 = 1.5$, $p = 0.2$. Epoch 2 vs. epoch 4: $t_5 = 1.8$, $p = 0.1$. Epoch 3 vs. epoch 4: $t_5 = 2.9$, $p = 0.035$.

For Fig. 4k. The difference of latency to transition between 10- and 5-second-long stimulation epochs during social contact. N = 6 (YFP), 6 (Con.), 6 (Stim.) mice. Unpaired t-test, adjusted $\alpha = 0.025$. YFP vs. Stim.: $t_{10} = 3.6$, $p = 0.005$. Con. vs. Stim.: $t_{10} = 3.4$, $p = 0.007$.

For Fig. 4l. Latency to transition during 10 s (epochs 1 and 3) or 5 s (epochs 2 and 4) optogenetic stimulation during novel object exploration. N = 6 (YFP), 6 (Con.), 6 (Stim.) mice. Unpaired t-test, adjusted $\alpha = 0.0063$. Epoch 1, YFP vs. Stim.: $t_9 = 5.6$, $p = 0.0003$. Con. vs. Stim.: $t_{10} = 4.9$, $p = 0.0007$. Epoch 2, YFP vs. Stim.: $t_9 = 8.7$, $p < 0.0001$. Con. vs. Stim.: $t_{10} = 6.2$, $p < 0.0001$. Epoch 3, YFP vs. Stim.: $t_{10} = 6.4$, $p < 0.0001$. Con. vs. Stim.: $t_{10} = 6.3$, $p < 0.0001$. Epoch 4, YFP vs. Stim.: $t_9 = 7.6$, $p < 0.0001$. Con. vs. Stim.: $t_{10} = 7.0$, $p < 0.0001$. For Stim., paired t-test, epoch 1 vs. epoch 2: $t_5 = 2.9$, $p= 0.033$. Epoch 1 vs. epoch 3: $t_5 = 1.2$, $p = 0.28$. Epoch 1 vs. epoch 4: $t_5 = 2.6$, $p = 0.05$. Epoch 2 vs. epoch 3: $t_5 = 4.0$, $p = 0.01$. Epoch 2 vs. epoch 4: $t_5 = 0.06$, $p = 0.95$. Epoch 3 vs. epoch 4: $t_5 = 4.3$, $p = 0.0076$.

For Fig. 4m. The difference of latency to transition between 10- and 5-second-long stimulation epochs during novel object exploration. N = 6 (YFP), 5 (Con.), 6 (Stim.) mice. Unpaired t-test, adjusted $\alpha = 0.025$. YFP vs. Stim.: $t_{10} = 3.8$, $p = 0.0036$. Con. vs. Stim.: $t_9 = 2.6$, $p = 0.0297$.

For Fig. 5a. *Left plot:* To estimate firing of multimodal LH cells according to the phase of beta oscillation during control epochs, we computed the firing probabilities of the same set of cells by randomly shuffling 2 s long windows in the whole recording sessions excluding epochs preceding transitions to all the scored behaviours. This procedure was repeated to obtain 1000 histograms with at least ten spikes for each cell. The firing probability in each bin was estimated as the mean across all shuffling sessions for each cell. Histograms in transition and control sets were then averaged between cells. *Right plot:* The preferred phase of each cell's phase distribution was computed for transition and for the 1000 reshuffling sessions. To compare these preferred phases distributions, we bootstrapped them 1000 times with replacement, fitted mixture of two circular distributions to the resulting distributions and estimated the ratio of cumulative probability within ±72° around the peak and within ±72° around the trough of the beta oscillation. We defined this preference as the peak/trough preference of the discharge of LH "transition" cells during transition to feeding vs. control epochs (n = 59 cells from four mice), unpaired t-test, $t_{1998} = 52.2$, $p < 0.0001$.

For Fig. 5b. *Left plot*: see Fig. 4a. *Right*: Peak/trough preference of the discharge of LH "transition" cells during transition to social contact vs. control epochs (n = 141 cells from five mice), unpaired t-test, $t_{1998} = 36.3$, $p < 0.0001$.

For Fig. 5d, **e**. To compare bivariate distributions of mean LH beta phases of simultaneously recorded LH and mPFC cells in transition and shuffled epochs, we performed a homogeneity test using likelihood ratio test under the null hypothesis stating that the distribution of mean LH beta phases do not differ for transition and shuffled epochs[73]. In this method, the probability density functions of bivariate circular distributions $\overline{\theta} = (\theta_1, \theta_2)$ are estimated by nonnegative trigonometric sums as follows:

$$f(\overline{\theta}; \overline{M}, \overline{c}) = \left\| \sum_{k_1=0}^{M_1} \sum_{k_2=0}^{M_2} c_{k_1 k_2} e^{i(k_1\theta_1 + k_2\theta_2)} \right\|^2 =$$

$$\sum_{k_1=0}^{M_1} \sum_{m_1=0}^{M_1} \sum_{k_2=0}^{M_2} \sum_{m_2=0}^{M_2} c_{k_1 k_2} \overline{c}_{m_1 m_2} e^{i[(k_1 - m_1)\theta_1 + (k_2 - m_2)\theta_2]},$$

$$s.t. \sum_{k=0}^{M_1} \sum_{m=0}^{M_2} \|c_{km}\|^2 = \left(\frac{1}{2\pi}\right)^2,$$

where the parameters $\overline{M} = (M_1, M_2)$correspond to the maximum number of modes in each of the two distribution, $\overline{c}$ contains the complex numbers $c_{k_1 k_2}$for $k_j = 0, \dots, M_j$ for $j = 1,2$, $\overline{c}_{m_1 m_2}$ refers to complex conjugate of $c_{m_1 m_2}$ and the space of $\overline{c}$ parameters corresponds to surface of a hypersphere. Thus, the density function of a bivariate circular distribution can be expressed as a mixture of multimodal circular distributions. To investigate the significance of the difference between the co-firing probability of simultaneously recorded LH and mPFC cells according to LH beta oscillation phase during transitions to feeding and shuffled transition to feeding (**d**), we considered different number of modes in the distributions, $\overline{M}$, all combinations up to $\overline{M} = (3,3)$. To perform the test for each $\overline{M}$, firstly we derived the maximum likelihood estimate of $\overline{c}$ parameters for combined data and individually for each of the transition and shuffled distributions. Then, we fitted the model (mixture of $\overline{M}$ circular distribution) to combined data, to transition and to shuffled distributions and obtained the maximum of the likelihood functions over the $\overline{c}$ parameters, for each of the three distributions. Finally, the test for homogeneity was constructed by comparing the maximum log likelihood for combined data to the sum of the maximum log likelihood for transition and shuffled distributions. The test statistics asymptotically followed a chi-squared distribution with $2(M_1 + 1)(M_2 + 1) - 2$ degree of freedom.

We repeated the test for different assumptions about the number of modes, $\overline{M}$, and obtained a distribution of p-values with the highest p-value equal to p = 0.0139 indicating the probability of the similarity of transition to feeding and shuffled transition epochs. Bayesian

information criterion was used for finding the maximum number of modes, $\overline{M}$, with best fit to each of the distributions. Thus, the null hypothesis, that the distributions of mean LH beta phases do not differ for transition to feeding and shuffled epochs, was rejected by p-value, $p < 0.0001$ (corresponding to the best cumulative fit).

Transitions to social contact vs. shuffled transitions to social contact epochs (**e**): (see above). Likelihood ratio test for homogeneity for different assumption about the parameter $\overline{M}$, all combinations up to $\overline{M} = (3{,}3)$, with highest p-value, $p < 0.0001$, and the representative p-value, $p < 0.0001$ for $\overline{M}$ with the best cumulative fit.

Proportion of coordinated vs. not coordinated mPFC and LH cell pairs (averaged across feeding and social contact) is significantly different during transition and control epochs. Chi-square test, $Chi^2 = 4.04$, $p = 0.04$.

For Fig. 6e. Optogenetic stimulation of mPFC-LH projections reduced the latency to behavioural transitions during feeding. N = 6 (YFP), 8 (Con.), 6 (Stim.) mice. Unpaired t-test, adjusted $\alpha = 0.025$. YFP vs. Stim.: $t_{10} = 3.6$, $p = 0.0048$. Con. vs. Stim.: $t_{12} = 6.2$, $p < 0.0001$.

For Fig. 6f. Optogenetic stimulation of mPFC-LH projections reduced the latency to behavioural transitions during social contact. N = 6 (YFP), 8 (Con.), 9 (Stim.) mice. Unpaired t-test, adjusted $\alpha = 0.0167$. YFP vs. Stim.: $t_{13} = 4.7$, $p = 0.0004$. Con. vs. Stim.: $t_{15} = 4.4$, $p = 0.0006$. Theta. vs. Stim.: $t_{15} = 4.9$, $p = 0.0002$.

For Fig. 6g. Optogenetic stimulation of mPFC-LH projections reduced the latency to behavioural transitions during novel object exploration. N = 6 (YFP), 8 (Con.), 8 (Stim.) mice. Unpaired t-test, adjusted $\alpha = 0.025$. YFP vs. Stim.: $t_{12} = 2.8$, $p = 0.015$. Con. vs. Stim.: $t_{14} = 3.4$, $p = 0.0042$.

For Fig. 6h. Effects of optogenetic excitation of mPFC-LPO projections on the latency to behavioural transitions during feeding. N = 6 (YFP), 8 (Con.), 7 (Stim.) mice. Unpaired t-test, adjusted $\alpha = 0.025$. YFP vs. Stim.: $t_{11} = 6.0$, $p < 0.0001$. Con. vs. Stim.: $t_{13} = 8.0$, $p < 0.0001$.

For Fig. 6i. Effects of optogenetic excitation of mPFC-LPO projections on the latency to behavioural transitions during social contact. N = 6 (YFP), 8 (Con.), 9 (Stim.) mice. Unpaired t-test, adjusted $\alpha = 0.025$. YFP vs. Stim.: $t_{13} = 0.03$, $p = 0.98$. Con. vs. Stim.: $t_{15} = 0.5$, $p = 0.64$.

For Fig. 6j. Effects of optogenetic excitation of mPFC-LPO projections on the latency to behavioural transitions during novel object exploration. N = 6 (YFP), 8 (Con.), 9 (Stim.) mice.

Unpaired t-test, adjusted α = 0.025. YFP vs. Stim.: t_{13} = 2.7, p = 0.018. Con. vs. Stim.: t_{15} = 2.5, p = 0.026.

For Fig. 7c. Beta oscillation amplitude during behavioural transitions, normalized to the amplitude in LPO. N = 4 mice. Unpaired t-test, adjusted α = 0.0167. LPO vs. LH: t_6 = 5.1, p = 0.002. LPO vs. VTA: t_6 = 13.1, p < 0.0001. LH vs. VTA: t_6 = 7.1, p = 0.0004.

For Fig. 7d. Beta oscillations coherence during transition to feeding (F), social contact (S) and novel object exploration (E). N = 4 mice. Repeated-measures two-way ANOVA (factors: brain region, transition type; repeated factor: animal), behaviour: $F_{2, 35}$ = 0.1, p = 0.9.
Multiple t-tests, adjusted α = 0.0167:
LPO-LH: F vs. S, t_6 = 0.2, p = 0.8. F vs. E, t_6 = 0.1, p = 0.9. S vs. E, t_6 = 0.3, p = 0.8.
LH-VTA: F vs. S, t_6 = 0.3, p = 0.8. F vs. E, t_6 = 0.08, p = 0.9. S vs. E, t_6 = 0.2, p = 0.8.
LPO-VTA: F vs. S, t_6 = 0.3, p = 0.8. F vs. E, t_6 = 0.02, p = 1.0. S vs. E, t_6 = 0.3, p = 0.8.
F: LPO-LH vs. LH-VTA, t_6 = 5.2, p = 0.002. LH-LPO vs. LPO-VTA, t_6 = 6.2, p = 0.0008. LH-VTA vs. LPO-VTA, t_6 = 1.1, p = 0.6.
S: LPO-LH vs. LH-VTA, t_6 = 5.1, p= 0.002. LH-LPO vs. LPO-VTA, t_6 = 6.2, p = 0.0008. LH-VTA vs. LPO-VTA, t_6 = 0.6, p = 0.6.
E: LPO-LH vs. LH-VTA, t_6 = 4.8, p = 0.003. LH-LPO vs. LPO-VTA, t_6 = 5.8, p = 0.001. LH-VTA vs. LPO-VTA, t_6 = 0.5, p = 0.6.

For Fig. 7f. The probability of tonic and burst discharge (interspike intervals >170 and <80 ms, respectively) of dopamine cells in relation to the maximum amplitude of VTA beta oscillation envelopes (n = 308 cells from three mice), highest probability within 100 ms lag. Paired t-test. Tonic vs. burst firing during beta, t_{307} = 12.8, p < 0.0001. Tonic vs. burst firing during 5 to 25 s shifted beta times (average across 500 shifts), t_{307} = 0.5, p = 0.6. Tonic firing, beta vs. shifted, t_{307} =3.4, p = 0.00065. Burst firing, beta vs. shifted, t_{307} = 3.0, p = 0.0026.

For Fig. 7g. Pearson correlation of match scores for different behaviours within individual 110 VTA cells: feeding and social contact, r = -0.03, p = 0.7; feeding and novel object expl., r = 0.04, p = 0.7; social contact and novel object expl., r = 0.17, p = 0.07.

For Fig. 7j. Decoding accuracy of behavioural transitions using individual behaviour phase signatures of dopamine cells in all phase bins. Permutation test for significance of the classifiers for each phase signature, p < 0.0001 (for details see Methods, Machine learning modelling).

For Extended Data Fig. 1c. Firing rate estimation of the LH population in relation to social contact and novel object exploration episodes, scaled for each cell from the maximum to the minimum. Firing rate curves were sorted in the ascending order of average values. The duration of episodes did not differ between consecutive sessions, one-way ANOVA, social contact: $F_{2,21}$ = 0.2, p = 0.8; object exploration: $F_{2,21}$ = 1.2, p = 0.3.

For Extended Data Fig. 1e. Cross-correlation between time stamps of the maximal amplitude of beta oscillation episodes and onsets of behaviours (reference), 2 sec baseline vs. 2 sec epochs upon behaviour onset, paired t-test: feeding, N = 7 mice, t_{12} = 0.08, p = 0.9; social contact, N = 8 mice, t_{14} = 0.11, p = 0.9; novel object exploration, N = 8 mice, t_{14} = 0.01, p = 1.0.

For Extended Data Fig. 1f. Average beta amplitude during each behaviour normalized within individual mice to the average beta amplitude during control epochs excluding these behaviours in seven (feeding) or eignt (social contact, novel object expl.) mice. One sampe t-test, Ho: The population mean equals 1, i.e. normalized amplitude of control epochs; feeding, t_{12} = 1.9, p = 0.09, social contact, t_{14} = 0.3, p = 0.8, novel obj.expl., t_{14} = 0.4, p = 0.7.

For Extended Data Fig. 2a. To verify whether in a sequence of behaviours transitions from one behaviour to another depend on the preceding behaviour we estimated the order of the time-homogeneous Markov chain probability model applied to recorded behavioural sequences for each mouse (eight mice). Markov chain models considered 17 behaviours (states) and featured orders ranging from 0 to 2 where order 0 corresponds to the independence between the behaviours, order 1 to the case when next behaviour depends only on the current behaviour and order 2 to the case when the next behaviour depends on the current and the preceding behaviours. The log likelihoods for Markov chains with above mentioned orders were computed independently for each session and then summed across sessions using the relative frequency of occurrences of behaviours. Then Bayesian Information Criterion (BIC) was derived to determine the best fitting model. The model of the order 0 (independence between the behaviours) had the lowest BIC value.

For Extended Data Fig. 2d. Average lag of syllables' usage change for transitions to feeding, social contact and novel object exploration; not different between syllables, one-way ANOVA, p = 0.12, $F_{2, 74}$ = 2.2. The average lag is a peak or trough (whichever is closer to the transition point) of the averaged across 9 mice normalized syllable usage curves.

For Extended Data Fig. 2i. The relative duration (fraction) of 2 sec transition epochs over the duration of a recording session, transitions to feeding: 0.014 ± 0.003, to social contact: 0.096 ± 0.011, to novel object expl.: 0.060 ± 0.012, mean ± SEM for eight mice.

For Extended Data Fig. 3b. The assessment of beta oscillation phase signatures during transition to feeding, social contact and novel object exploration (combined) for LH populations specific for these bahaviours. Each phase signature was computed for a population of cells with match scores in lower or upper ten percentiles. For accurate estimation of match scores distributions, the latter distributions were subsampled with replacement (bootstrapped) as many times as needed to obtain the same number of cells as in the respective complete population. SVM accuracies of decoding transition to the three behaviours (combined) using phase signatures of individual behaviours for a more selective LH population in eight phase bins (mean ± SEM), φ_F : 78 ± 5%, φ_S : 84 ± 2%, φ_E: 88 ± 4%, and using phase singnatures of multiple behaviours, 94 ± 3%. The comparison of decoding accuracies using phase signatures to individual behaviours and their combination, n = 8 phase bins, paired-test, adjusted α = 0.0167, φ_F vs. All, $t_7 = 4.75$, p = 0.002, φ_S vs. All, $t_7 = 4.51$, p = 0.0027, φ_E vs. All, $t_7 = 3.28$, p = 0.0133.

For Extended Data Fig. 3f. Ho: peak/trough preference of the average phase signature is equal or less than 1 (a uniform phase signature), bootstrap test, adjusted α = 0.03, transition: p = 0.42, control epochs: p = 0.43.

For Extended Data Fig. 4d. Randomization test, probability of observing the decoding accuracies (averaged across F, S, E phase signatures) in the 1000 phase-shuffled datasets equal or greater than the decoding accuracy in the original dataset, p = 0.0009.

For Extended Data Fig. 4e. Unpaired t-test, φ_F: $t_4 = 15.90$, $p < 0.0001$, φ_S: $t_4 = 3.14$, p = 0.035, φ_E: $t_4 = 11.73$, p = 0.0003.

For Extended Data Fig. 4i. Accuracies of decoding transitions to the three behaviours (combined) using individual behaviour phase signatures of 157 beta phase-locked vs. 514 non-modulated LH cells, n = 8 bins. Paired t-test, φ_F: $t_7 = 1.0$, p = 0.33, φ_S: $t_7 = 1.5$, p = 0.17, φ_E: $t_7 = 1.3$, p = 0.23.

For Extended Data Fig. 4j. Accuracies of decoding transitions to the three behaviours (combined) using multiple behaviour phase signatures of beta phase-locked vs. non-modulated LH cells, n = 8 phase bins. Paired t-test, $t_7 = 1.1$, p = 0.32. Accuracies for SVMs

using phase signatures related to individual vs. multiple (All) behaviours, n = 8 phase bins, paired t-test, adjusted α = 0.0167, phase-locked cells: φ_F vs. All, t_7 = 2.4, p = 0.0465, φ_S vs. All, t_7 = 4.0, p = 0.0046, φ_E vs. All, t_7 = 5.7, p = 0.0007. Non-modulated cells: φ_F vs. All, t_7 = 5.1, p = 0.0013, φ_S vs. All, t_7 = 5.9, p = 0.0005, φ_E vs. All, t_7 = 4.5, p = 0.0028.

For Extended Data Fig. 5b. The accuracy of decoding upcoming behaviour using novel object exploration phase signatures (φ_F) in eight phase bins in the peak neighborhood, computed during transitions, fed to the same SVM model: 82%. The accuracy of decoding current behaviour using φ_F in the peak neighborhood, computed during 2-s random epochs during feeding, social contact or novel object exploration, fed to the same SVM model: 40%, chance level 33.3%. Permutation test for significance of the classifiers decoding upcoming behaviour: p < 0.0001, decoding current behaviour: p = 0.0004 (for details see Methods, Machine learning modelling).

For Extended Data Fig. 6f. Firing rate of 44 LH cells from one mouse during baseline vs. beta in-phase stimulation. Wilcoxon signed-rank test, Z = 1.9, p = 0.054.

For Extended Data Fig. 6g. Firing rate of LH and LPO cells (LH: n = 255 cells, LPO cells: n = 106 cells from two mice) during baseline, beta out-of-phase stimulation protocol and non-rhythmic control stimulation protocol. Two-way ANOVA (factors: brain region, stimulation protocol), stimulation protocol: $F_{2,\ 1013}$ = 1.01, p = 0.4.

For Extended Data Fig. 6h. Accuracies of decoding transitions from feeding, social contact and novel object exploration (combined) to any of the scored behaviours vs. control non-transition epochs using peak neighbourhood phase signatures of the multiple behaviours, p < 0.0001 in each permutation test for significance of the classifiers computed in individual phase bins (for details see Methods**,** Machine learning modelling).

For Extended Data Fig. 6j. Latency to transition from feeding, social contact and novel object exploration during stimulation (10 and 5 sec light on epochs combined) applied upon respective behaviours' onset and during light off epochs. Unpaired t-tests. Feeding: N = 6 (On), 5 (Off) mice, On vs. Off, t_9 = 11.9, p < 0.0001. Social contact: N = 6 (On), 6 (Off) mice, On vs. Off, t_{10} = 9.5, p < 0.0001. Novel object exploration: N = 6 (On), 6 (Off) mice, On vs. Off, t_{10} = 7.2, p < 0.0001.

For Extended Data Fig. 6k. Average speed during beta out-of-phase vs. non-rhythmic optogenetic stimulation. N = 5 (Con.), 5 (Stim.) mice. Unpaired t-test, t_8 = 0.4, p = 0.7.

For Extended Data Fig. 6l. Path length during beta out-of-phase vs. non-rhythmic optogenetic stimulation. N = 5 (Con.), 5 (Stim.) mice. Unpaired t-test, $t_8 = 0.2$, $p = 0.9$.

For Extended Data Fig. 6m. The fraction of chasing over number of social contact episodes during 10 s optogenetic stimulation. N = 6 (YFP), 6 (Con.), 5 (Stim.) mice. Unpaired t-test, adjusted $\alpha = 0.025$. Stim.: N = 5 mice. YFP vs. Stim.: $t_9 = 16.2$, $p < 0.0001$. Con. vs. Stim.: $t_9 = 15.1$, $p < 0.0001$.

For Extended Data Fig. 6n. Latency to feeding (F), social contact (S) or novel object exploration (E) (combined) during beta out-of-phase and control (YFP) stimulation upon social contact onset. N = 4 (YFP), 6 (Stim.). Unpaired t-test, $t_8 = 3.54$, $p = 0.0076$.

For Extended Data Fig. 6o. Comparison of cumulative distribution of the latencies to transition during out-of-phase stimulation in the absence of an ongoing behaviour vs. non-rhythmic stimulation, two-sample KS-test, $D = 0.7$, $p = 0.0002$.

For Extended Data Fig. 6p. Latency to feeding, social contact or novel object exploration (combined) during stimulation in the absence of these behaviours. N = 6 (YFP), 6 (Con.), 6 (Stim.) mice. Unpaired t-tests, adjusted $\alpha = 0.025$. Stim. vs. YFP: $t_{10} = 2.5$, $p = 0.03$. Stim. vs. Con., $t_{10} = 3.0$, $p = 0.01$.

For Extended Data Fig. 6q. A total duration of feeding during one-way (LPO-LH) or out-of-phase (Stim.) stimulation (20 min stimulation, delivered non-contingent on behaviours). N = 6 (LPO-LH), 5 (Stim.) mice. Unpaired t-test, $t_9 = 4.6$, $p = 0.0014$.

For Extended Data Fig. 6r. A total duration of social contact during one-way (LPO-LH) or out-of-phase (Stim.) stimulation (20 min stimulation, delivered non-contingent on behaviours). N = 6 (LPO-LH), 6 (Stim.) mice. Unpaired t-test, $t_{10} = 2.3$, $p = 0.042$.

For Extended Data Fig. 7a. Comparison of peak/trough preference of phase signature for each behaviour vs. 1 - a uniform phase signature, one-sample t-test (right tailed). Feeding: $t_{999} = 16.2$, $p < 0.0001$. Social contact: $t_{999} = -6.4$, $p = 1$. Novel object exploration: $t_{999} = -101.7$, $p = 1$.

For Extended Data Fig. 7c, Latency to feeding and the probability of feeding onset during. N = 6 (YFP), 4 (In-phase) mice. Unpaired t-tests. In-phase vs. YFP, latency, $t_8 = 2.4$, $p = 0.043$;

probability of onset, $t_8 = 5.2$, $p = 0.0008$.

For Extended Data Fig. 7d, Latency to social contact. N = 5 (YFP), 5 (In-phase) mice. Unpaired t-test. In-phase vs. YFP, latency, $t_8 = 0.6$, $p = 0.6$.

For Extended Data Fig. 7e, Latency to novel object exploration. N = 6 (YFP), 5 (In-phase) mice. Unpaired t-test. In-phase vs. YFP, latency, $t_9 = 1.3$, $p = 0.2$.

For Extended Data Fig. 7g, Latency to transition during 10 s or 5 s in-phase LPO-LH stimulation during feeding (F), social contact (S) and novel object exploration (E). N = 5 mice. Unpaired t-tests, adjusted $\alpha = 0.0063$. Epoch 1, F vs. S: $t_7 = 6.8$, $p = 0.0003$. F vs. E: $t_7 = 5.6$, $p=0.0008$. Epoch 2, F vs. S: $t_8 = 9.1$, $p < 0.0001$. F vs. E: $t_8 = 6.3$, $p = 0.0002$. Epoch 3, F vs. S: $t_7 = 4.9$, $p = 0.0018$. F vs. E: $t_8 = 3.8$, $p = 0.0052$. Epoch 4, F vs. S: $t_8 = 3.9$, $p = 0.0045$. F vs. E: $t_8 = 4.7$, $p = 0.0015$. For Feeding, paired t-tests, epoch 1 vs. epoch 2: $t_4 = 3.0$, $p = 0.04$. Epoch 1 vs. epoch 3: $t_4 = 1.9$, $p = 1.3$. Epoch 1 vs. epoch 4: $t_4 = 3.9$, $p = 0.017$. Epoch 2 vs. epoch 3: $t_4 = 1.4$, $p = 0.25$. Epoch 2 vs. epoch 4: $t_4 = 1.2$, $p = 0.3$. Epoch 3 vs. epoch 4: $t_4 = 1.7$, $p = 0.17$.

For Extended Data Fig. 7h, The difference of latency to transition between 10 s and 5 s stimulation during feeding, social contact and novel object exploration. N = 5 mice. Unpaired t-test, adjusted $\alpha = 0.025$. F vs. S: $t_8 = 3.1$, $p = 0.015$. F. vs. E: $t_8 = 3.0$, $p = 0.017$.

For Extended Data Fig. 8a. Right: Peak/trough preference of the discharge of LH multimodal cells during transition to novel object exploration vs. control epochs (n = 110 cells from three mice), unpaired t-test, $t_{999} = 0.5$, $p = 0.59$.

For Extended Data Fig. 8b. Comparison of spectral coherence of the LFP in the mPFC and the LH during transitions and control epochs, excluding transitions, in the beta frequency band, N = 3 mice, repeated measure two-way ANOVA (factors: type of transition (upcoming behaviours or control epochs), frequency in the beta band, repeated factor: animal): simple main effect of the type of transition, $F_{4,120} = 0.78$, $p = 0.5$. Coherence in beta and gamma (30-60 Hz) bands (factor 1) and different types of transitions (factor 2), two-way ANOVA, simple main effect of the frequency band: $F_{1,20} = 115.3$, $p < 0.0001$, of the type of transition: $F_{4,20} = 0.4$, $p = 0.8$.

For Extended Data Fig. 8d. Beta oscillations coherence during complete recording sessions between mPFC and LH (N = 3 mice) and BLA/CeA and LH (N = 4 mice). Unpaired t-test, t_5 = 8.9, p = 0.0003.

For Extended Data Fig. 8h-j. Pearson's correlations of the mPFC cells' population firing probability according to the LH beta phase between epochs prior to transitions, during transitions, and after behaviour onset. Between transition and prior to transition, r = -0.11, p = 0.6; between transition and behaviour, r = -0.29, p = 0.2; between prior to transition and behaviour, r = 0.66, p = 0.0015.

For Extended Data Fig. 9e. Average speed during optogenetic excitation of mPFC-LH projections. N = 6 (YFP), 9 (Stim.) mice. Unpaired t-test, t_{13} = 2.0, p = 0.07.

For Extended Data Fig. 9f. Path length during optogenetic excitation of mPFC-LH projections. N = 6 (YFP), 9 (Stim.) mice. Unpaired t-test, t_{13} = 1.4, p = 0.2.

For Extended Data Fig. 9g. Effects of optogenetic inhibition of mPFC-LH projections on the latency to behavioural transitions during social contact. N = 6 (YFP), 9 (Inhib.) mice. Unpaired t-test, t_{13} = 3.6, p = 0.003.

For Extended Data Fig. 9h. Effects of optogenetic inhibition of mPFC-LH projections on the latency to behavioural transitions during novel object exploration. N = 7 (YFP), 9 (Inhib.) mice. Unpaired t-test, t_{14} = 3.2, p = 0.006.

For Extended Data Fig. 9i. Effects of optogenetic inhibition of mPFC-LH projections on the latency to behavioural transitions during feeding. N = 7 (YFP), 8 (Inhib.) mice. Unpaired t-test, t_{13} = 0.2, p = 0.9.

For Extended Data Fig. 9j. Average speed during optogenetic excitation of mPFC-LPO projections. N = 6 (YFP), 9 (Stim.) mice. Unpaired t-test, t_{13} = 0.5, p = 0.6.

For Extended Data Fig. 9k. Path length during optogenetic excitation of mPFC-LPO projections. N = 6 (Con.), 9 (Stim.) mice. Unpaired t-test, t_{13} = 1.9, p = 0.08.

For Extended Data Fig. 9l. Effects of optogenetic inhibition of mPFC-LPO projections on the latency to behavioural transitions during social contact. N = 7 (YFP), 9 (Inhib.) mice. Unpaired t-test, t_{14} = 0.1, p = 0.9.

For Extended Data Fig. 9m. Effects of optogenetic inhibition of mPFC-LPO projections on the latency to behavioural transitions during novel object exploration. N = 7 (YFP), 9 (Inhib.) mice. Unpaired t-test, $t_{14} = 1.8$, $p = 0.09$.

For Extended Data Fig. 9n. Effects of optogenetic inhibition of mPFC-LPO projections on the latency to behavioural transitions during feeding. N = 5 (YFP), 9 (Inhib.) mice. Mann-Whitney test, $p = 0.002$.
