# Supplementary material for: The dynamic state of a prefrontal–hypothalamic–midbrain circuit commands behavioral transitions
Source: Nat Neurosci. 2024 Mar 18;27(5):952–63. doi: 10.1038/s41593-024-01598-3 (PMC11089001; doi:10.1038/s41593-024-01598-3)
Supplement: Supplementary file 2 — Reporting Summary [file 41593_2024_1598_MOESM2_ESM.pdf]

nature portfolio

Corresponding author(s): T. Korotkova, A. Ponomarenko

Last updated by author(s): Jan 23, 2024

Reporting Summary

Nature Portfolio wishes to improve the reproducibility of the work that we publish. This form provides structure for consistency and transparency in reporting. For further information on Nature Portfolio policies, see our Editorial Policies and the Editorial Policy Checklist.

Statistics

For all statistical analyses, confirm that the following items are present in the figure legend, table legend, main text, or Methods section.

n/a	Confirmed	
☐	☒	The exact sample size (*n*) for each experimental group/condition, given as a discrete number and unit of measurement
☐	☒	A statement on whether measurements were taken from distinct samples or whether the same sample was measured repeatedly
☐	☒	The statistical test(s) used AND whether they are one- or two-sided<br>*Only common tests should be described solely by name; describe more complex techniques in the Methods section.*
☐	☒	A description of all covariates tested
☐	☒	A description of any assumptions or corrections, such as tests of normality and adjustment for multiple comparisons
☐	☒	A full description of the statistical parameters including central tendency (e.g. means) or other basic estimates (e.g. regression coefficient) AND variation (e.g. standard deviation) or associated estimates of uncertainty (e.g. confidence intervals)
☐	☒	For null hypothesis testing, the test statistic (e.g. *F*, *t*, *r*) with confidence intervals, effect sizes, degrees of freedom and *P* value noted<br>*Give P values as exact values whenever suitable.*
☒	☐	For Bayesian analysis, information on the choice of priors and Markov chain Monte Carlo settings
☒	☐	For hierarchical and complex designs, identification of the appropriate level for tests and full reporting of outcomes
☐	☒	Estimates of effect sizes (e.g. Cohen's *d*, Pearson's *r*), indicating how they were calculated

Our web collection on statistics for biologists contains articles on many of the points above.

Software and code

Policy information about availability of computer code

Data collection	Neuralynx Cheetah Data Acquistion software for electrophysiological data,<br>Motif video recording software for a part of behavioural data,<br>Leica Application Suite X (LAS X) software for confocal imaging,<br>Zeiss Imager microscope (Axio Imager M2 Microscope, Zeiss, Oberkochen, Germany) for wide-field imaging.
Data analysis	GraphPad Prism 9 (GraphPad Software) for plotting figures and statistical analysis<br>Fiji - ImageJ for analysing imaging<br>Neurophysiological Data Manager (NDManager, http://neurosuite.sourceforge.net) for preprocessing of electrophysiological data<br>Klustakwik (http://klusta-team.github.io/klustakwik/) for spike sorting<br>Adobe Premiere Pro 2020 for video rendering<br>Adobe Illustrator 2020 for assembling figures<br>Matlab 2014b (Mathworks) for analysing electrophysiological data, statistical analysis and behaviors<br>Python 3 for machine learning modelling data analysis and statistical analysis<br>Scikit-learn package (version 1.2.2)<br>DeepLabCut toolbox (version 2.2.0.2)<br>All codes used in the current study are available via GitHub (https://github.com/NeuroAnalyz/SpikeTrains-LFP) or from corresponding authors upon reasonable request.

For manuscripts utilizing custom algorithms or software that are central to the research but not yet described in published literature, software must be made available to editors and reviewers. We strongly encourage code deposition in a community repository (e.g. GitHub). See the Nature Portfolio guidelines for submitting code & software for further information.

Data

Policy information about availability of data

All manuscripts must include a data availability statement. This statement should provide the following information, where applicable:

- Accession codes, unique identifiers, or web links for publicly available datasets
- A description of any restrictions on data availability
- For clinical datasets or third party data, please ensure that the statement adheres to our policy

Spike trains recorded in the LH, mPFC, VTA and timestamps of beta oscillations were made available via Figshare (https://doi.org/10.6084/m9.figshare.22317091). Further datasets generated during the current study are available from corresponding authors upon reasonable request.

Human research participants

Policy information about studies involving human research participants and Sex and Gender in Research.

Reporting on sex and gender	n/a
Population characteristics	n/a
Recruitment	n/a
Ethics oversight	n/a

Note that full information on the approval of the study protocol must also be provided in the manuscript.

Field-specific reporting

Please select the one below that is the best fit for your research. If you are not sure, read the appropriate sections before making your selection.

☒ Life sciences ☐ Behavioural & social sciences ☐ Ecological, evolutionary & environmental sciences

For a reference copy of the document with all sections, see nature.com/documents/nr-reporting-summary-flat.pdf

Life sciences study design

All studies must disclose on these points even when the disclosure is negative.

Sample size	Sample size was chosen using our experience and standards in the field (citations are included). Reported in Methods, Electrophysiological data analysis, Machine learning modelling and Statistical analysis.
Data exclusions	Grubbs' test was used to exclude outlier points from behavioural datasets. Median absolute deviation outlier test was used to exclude outlier points from the analysis of optogenetic entrainment. Reported in Methods, Statistical analysis.
Replication	All attempts at replication were successful. Reported in figure legends.
Randomization	Animals were randomly assigned to control and experimental groups.
Blinding	Computations were subsequently performed blindly using automatic selection of data from a database.

Reporting for specific materials, systems and methods

We require information from authors about some types of materials, experimental systems and methods used in many studies. Here, indicate whether each material, system or method listed is relevant to your study. If you are not sure if a list item applies to your research, read the appropriate section before selecting a response.

Materials & experimental systems

n/a	Involved in the study
☒	☐ Antibodies
☒	☐ Eukaryotic cell lines
☒	☐ Palaeontology and archaeology
☐	☒ Animals and other organisms
☒	☐ Clinical data
☒	☐ Dual use research of concern

Methods

n/a	Involved in the study
☒	☐ ChIP-seq
☒	☐ Flow cytometry
☒	☐ MRI-based neuroimaging

Animals and other research organisms

Policy information about studies involving animals; ARRIVE guidelines recommended for reporting animal research, and Sex and Gender in Research

Laboratory animals	Vgat-ires-Cre knock-in mice (The Jackson Laboratory, Bar Harbour, USA) and C57BL/6 mice, 10 - 25 weeks old, were used.
Wild animals	No wild animals were used this study.
Reporting on sex	Male and female mice were used in this study.
Field-collected samples	No field collected samples were used in this study.
Ethics oversight	All animal procedures were performed in accordance with national and international guidelines and were approved by the local health authority (LANUV, Das Landesamt für Natur, Umwelt und Verbraucherschutz).

Note that full information on the approval of the study protocol must also be provided in the manuscript.
